# Supplementary material for: Cenozoic aridization in Central Eurasia shaped diversification of toad-headed agamas (Phrynocephalus; Agamidae, Reptilia)
Source: PeerJ. 2018 Mar 19;6:e4543. doi: 10.7717/peerj.4543 (PMC5863718; doi:10.7717/peerj.4543)
Supplement: Supplemental Information 1 [file peerj-06-4543-s001.docx]

**Supplementary File 1. Review on history of phylogenetic studies of the genus *Phrynocephalus*.**

Bedriaga (1907; 1909; 1912) and Tsarevsky (1926; 1927; 1929) first attempted to provide phylogenetic hypotheses for *Phyrnocephalus*. However, these were quite speculative and based on largely homoplastic traits of external morphology. Thus, the taxonomy of the group remains problematic. Cranial morphology in scope of phylogenetic relationships was discussed by Tsarevsky (1964) and Dunayev (1993). The unpublished thesis of Whiteman (1978) provided an important breakthrough in studies of the evolutionary history of the genus. This work was based on a cladistic analysis of 16 osteological characters for 23 species. Unfortunately, much of the results are tentative due to specimen misidentification and inappropriate outgroup taxa.

Arnold (1999) pursued the evolutionary history of 25 species of *Phrynocephalus* based on cladistic analysis of 46 characters of external morphology, anatomy and osteology. His phylogeny largely contradicted that of Whiteman (1978). Dunayev (1996) provided an evolutionary hypothesis for 12 Central Asian species of *Phrynocephalus* based on analysis of ethological features, including seven characters of tail signaling. All researchers agreed that *Phrynocephalus* was monophyletic, but its position among other agamids remained controversial. The morphological analysis of Moody (1980) indicated that *Phrynocephalus* was derived from a paraphyletic *Agama* s. stricto, whereas results of isozyme analysis by Ananjeva and Sokolova (1990) suggested that *Phrynocephalus* was the sister-group of *Laudakia*. Simultaneously, Arnold (1999) argued that genus *Trapelus* formed a sister-group to the two toad-headed genera *Phrynocephalus* and *Bufoniceps*. The subsequent molecular analysis of Macey *et al.* (2006) placed *Phrynocephalus* as the sister taxon to *Trapelus* Cuvier 1816.

The first use of DNA sequence data to *Phrynocephalus* were made by Pang *et al.* (2003), who studied the phylogenetic relationships of ca. 16 Chinese taxa based on four mtDNA gene fragments. They identified two major clades: the Tibetan viviparous group and an oviparous group. Monophyly of the former was highly supported and the authors implied that uplifting of the Qinghai-Tibetan Plateau played a fundamental role in the diversification of *Phrynocephalus*. Regardless, their analyses did not resolve many of the interrelationships among the oviparous species. Wang and Fu (2004) addressed some questions concerning the taxonomy of Chinese oviparous species of the *P. versicolor* species complex (*P. versicolor*, *P. frontalis* and *P. przewalskii*) based on *ND2* sequences, although the broader evolutionary and biogeographic questions remained unclear. Later, Guo and Wang (2007) reanalyzed the mtDNA sequence data of Pang *et al.* (2003) and applied Bayesian divergence-time estimation, suggesting that *Phrynocephalus* originated at the Middle–Late Miocene boundary (15.16–10.4 Ma). Solovyeva *et al.* provided phylogenetic analysis of *P. helioscopus* species complex based on mtDNA and nuDNA data (Solovyeva *et al.* 2011) and morphological analysis (Solovyeva *et al.* 2012). Recently, Melville *et al.* (2009) provided an important contribution to understanding phylogenetic relationships of *Phrynocepahlus* by analyzing mtDNA and nuDNA sequences data for seven Middle Asian species, including phylogenetically distant *P. interscapularis* Fitzinger, 1826, and published data for five Central Asian taxa. Their results confirmed sister-clade relationships of *Phrynocephalus* and *Laudakia* and estimated the basal differentiation within the genus *Phrynocephalus* took place in the late Oligocene (28.9 Ma).

**References:**

Ananjeva NB, Sokolova TM. 1990. The position of the genus *Phrynocephalus* Kaup 1825 in agamids system. *Trudy Zool. Inst. Akad. Nauk SSSR* 207:12–21. [in Russian]

Arnold EN. 1999. Phylogenetic relationships of toad-headed lizards (*Phrynocephalus*, Agamidae) based on morphology. *Bulletin of the British Museum (Natural History), Zoology* 65(1,З):1–13.

Bedriaga J. 1907. Amphibien und Reptilien (Reptilia Przewalskiana). Wissenschaftliche Resultate der von N.M. Przewalski nach Central-Asien unternommenen Reisen. *Zoologischer Theil Bd*. 3 Abth. 1 Lfrg. 2:71–278.

Bedriaga J. 1909. Amphibien und Reptilien (Reptilia Przewalskiana). Wissenschaftliche Resultate der von N.M. Przewalski nach Central-Asien unternommenen Reisen. *Zoologischer Theil Bd*. 3 Abth. 1 Lfrg. 3:279–502.

Bedriaga J. 1912 Amphibien und Reptilien (Reptilia Przewalskiana). Wissenschaftliche Resultate der von NM Przewalski nach Central-Asien unternommenen Reisen. *Zoologischer Theil Bd*. 3 Abth. 1 Lfrg. 4:503–769.

Dunayev EA. 1993. The cranial morphology and phylogeny of *Prynocephalus* species. *7th Ordinary General Meeting Societas Europaea Herpetologica*, Barcelona, 62.

Dunayev EA. 1996 On the possible use of the ethological features in the taxonomy and phylogeny of toad agamas. *Russian Journal of Herpetology* 3(1):32–38.

Guo X, Wang Y. 2007. Partitioned Bayesian analyses, dispersal-vicariance analysis, and the biogeography of Chinese toad-headed lizards (Agamidae: *Phrynocephalus*): A re-evaluation. *Molecular Phylogenetics and Evolution* 45(2):643–662. https://doi.org/10.1016/j.ympev.2007.06.013

Macey JR, Schulte II JA, Fong JJ, Das I, Papenfuss TJ. 2006. The complete mitochondrial genome of an agamid lizard from the Afro-Asian subfamily Agaminae and the phylogenetic position of *Bufoniceps* and *Xenagama*. *Molecular Phylogenetics and Evolution* 39:291–297. https://doi.org/10.1016/j.ympev.2005.08.020

Melville J, Hale J, Mantziou G, Ananjeva NB, Milto K, Clemann N. 2009. Historical biogeography, phylogenetic relationships and intraspecific diversity of agamid lizards in the Central Asian deserts of Kazakhstan and Uzbekistan. *Molecular Phylogenetics and Evolution* 53:99–112. https://doi.org/10.1016/j.ympev.2009.05.011

Pang J, Wang Y, Zhong Y, Hoelzele AR, Papenfuss TJ, Zeng X, Ananjeva NB, Zhang YP. 2003. A phylogeny of Chinese species in the genus *Phrynocephalus* (Agamidae) inferred from mitochondrial DNA sequences. *Molecular Phylogenetics and Evolution* 27:398–409.

Solovyeva EN, Dunayev EA, Poyarkov NA. 2012. Interspecific taxonomy of sunwatcher toadhead agama species complex (*Phrynocephalus helioscopus*, Squamata). *Zoologicheskiy Zhurnal* 91(11):1377–1396. [in Russian]

Solovyeva EN, Poyarkov NA, Dunayev EA, Duysebayeva TN, Bannikova AA. 2011. Molecular differentiation and taxonomy of the sunwatcher toad headed agama species complex *Phrynocephalus* superspecies *helioscopus* (Pallas 1771) (Reptilia: Agamidae). *Russian Journal of Genetics* 47(7):952–967.

Tsarevsky SF. 1926. Contributions to the systematics and dispersal of lizards of the genus *Phrynocephalus* (Reptilia). *Doklady AN SSSR* 119–122. [in Russian]

Tsarevsky SF. 1927. About the taxonomy and distribution of lizards of the *Phrynocephalus* genus. *Doklady AN SSSR* A(2):23–26. [in Russian]

Tsarevsky SF. 1929. Contributions to the systematics and dispersal of lizards of the genus *Phrynocephalus* (Reptilia). *Doklady AN SSSR* A(17):415–419. [in Russian]

Tsarevsky SF. 1964. Towards systematics of the genus *Phrynocephalus*. *In*: *Voprosy Gerpetologii, Abstracts of the 1st All-Union Herpetology Conference*, Leningrad, 74–75. [in Russian]

Wang Y, Fu J. 2004. Cladogenesis and vicariance patterns in the toad-headed lizard *Phrynocephalus versicolor* species complex. *Copeia* 2:199–206. https://doi.org/10.1643/CG-03-082R1

Whiteman RS. 1978. Evolutionary history of the lizard genus *Phrynocephalus* (Lacertilia, Agamidae). M.A. Thesis, Fullerton, California State University 113.
