# Supplementary material for: Cenozoic aridization in Central Eurasia shaped diversification of toad-headed agamas (Phrynocephalus; Agamidae, Reptilia)
Source: PeerJ. 2018 Mar 19;6:e4543. doi: 10.7717/peerj.4543 (PMC5863718; doi:10.7717/peerj.4543)
Supplement: Supplemental Information 2 [file peerj-06-4543-s002.docx]

**Supplementary File 2. Protocols of DNA PCR amplification used in this study.**

***Protocol of amplification for RAG-1* (as in Shoo *et al.* 2008 with modifications):**

1. Denaturation at 95 ºС 15 min.

2.1. Denaturation at 95 ºС 30 s.

2.2. Annealing at 70 ºС 20 s (touchdown 2 ºС every cycle).

2.3. Elongation at 72 ºС 1 min 30 s.

Х 9

3.1. Denaturation at 95 ºС 30 s.

3.2. Annealing at 70 ºС 30 s.

3.3. Elongation at 72 ºС 45 s.

X 36

4. Additional elongation at 72 ºС 4 min.

***Protocol of amplification for BDNF* (Townsend *et al.* 2008):**

1. Denaturation at 94 ºС 3 min.

2.1. Denaturation at 94 ºС 30 s.

2.2. Annealing at 62 ºС 1 min.

2.3. Elongation at 72 ºС 1 min.

Х 36

3. Additional elongation at 72 ºС 6 min.

***Protocol of amplification for AKAP9, NKTR* (as in Townsend *et al.* 2011 with modifications):**

1. Denaturation at 95 ºС 4 min.

2.1. Denaturation at 95 ºС 30 s.

2.2. Annealing at 60 ºС for AKAP9 and at 68 ºС for NKTR 45 s. (touchdown every cycle for 0,7 ºС for AKAP9 and for 1 ºС for NKTR).

2.3. Elongation at 72 ºС 1 min.

Х 20

3.1. Denaturation at 95 ºС 30 s.

3.2. Annealing at 46 ºС 45 s.

3.3. Elongation at 72 ºС 1 min.

4. Additional elongation at 72 ºС 7 min.

5. Additional elongation at 60 ºС 40 min.

**References:**

Shoo L, Rose R, Doughty P, Austin JJ, Melville J. 2008. Diversification patterns of pebble-mimic dragons are consistent with historical disruption of important habitat corridors in arid Australia. *Molecular Phylogenetics and Evolution* 48:528–542. https://doi.org/10.1016/j.ympev.2008.03.022

Townsend TM, Alegre RE, Kelley ST, Wiens JJ, Reeder TW. 2008. Rapid development of multiple nuclear loci for phylogenetic analysis using genomic resources: an example from squamate reptiles. *Molecular Phylogenetics and Evolution* 47(1):129–142. https://doi.org/10.1016/j.ympev.2008.01.008.

Townsend TM, Mulcahy DG, Noonan BP, Sites JW, Kuczynski CA, Wiens JJ, Reeder TW. 2011. Phylogeny of iguanian lizards inferred from 29 nuclear loci, and a comparison of concatenated and species-tree approaches for an ancient, rapid radiation. *Molecular Phylogenetics and Evolution* 61(2011):363–380. https://doi.org/10.1016/j.ympev.2011.07.008
