# Supplementary material for: Cenozoic aridization in Central Eurasia shaped diversification of toad-headed agamas (Phrynocephalus; Agamidae, Reptilia)
Source: PeerJ. 2018 Mar 19;6:e4543. doi: 10.7717/peerj.4543 (PMC5863718; doi:10.7717/peerj.4543)
Supplement: Supplemental Information 3 [file peerj-06-4543-s003.docx]

**Supplementary File 3. Biogeographic area definition for Central Eurasia.**

In the present paper follow the Soviet/Russian (bio)geographic tradition which divides the Central Eurasia into “Central Asia” and “Middle Asia” as separate biogeographic units (whereas they are known simply as “Central Asia” in the western (bio)geographic tradition). According to these views, “Central Asia” encompasses arid areas of China, Mongolia and southern Siberia, including Tibet (Xizang) and western China (Xinjiang), whereas areas west from Pamir-Alai – Tien Shan – Junggar Alatau – Altai Mountains belong to “Middle Asia”. “Middle Asian” area includes steppes and deserts of modern Kazakhstan, northern Caspian Basin and Ciscaucasia, and the territory of the former Soviet Middle Asian Republics: Uzbekistan, Turkmenistan, Kyrgyzstan and Tajikistan. “Middle Asian” and “Central Asian” faunas are isolated from each other by a massive mountainous belt and represent distinct biogeographic entities.

For subdivision of Central Eurasia into discrete biogeographic regions for paleogeographic reconstruction we followed major works on biogeography of Central Eurasia (Geptner 1938; Korovin 1961) and on biogeographic analysis of Eurasian herpetofauna (Nikolsky 1915; Chernov 1949; Bogdanov 1965; Chkhikvadze et al. 1983; Rustamov & Szczerbak 1985; Ananjeva & Tuniyev, 1992; Sindaco & Jeremcenko 2008).

In total, within the Central Eurasia we defined seven regions for the analyses:

1. **Northern Central Asia (CA):** arid areas of western and central China, Mongolia and southern Siberia (Russian Federation) eastwards from Pamir-Alai, Tien Shan and Junggar Alatau Mts., northwards from Karakoram, Kun Lun and Altun Shan Mts. in central China, southwards from Altai and Sayan Mts. in southern Siberia.
2. **Qinhai –** **Tibetan Plateau** **(TI)**: high elevation arid areas in Central China southwards from Kun Lun and Altun Shan Mts., northwards from main Himalaya Mountain Ridge, westwards bordered by Karakoram Mountains.
3. **Kazakhstan, North Caspian and Ciscaucasian deserts (KZ):** arid lowland areas of Kazakhstan, Kyrgyzstan and southern Russia westwards from Altai, Junggar Alatau and Tien Shan Mts., southwards from steppe/desert border in southern Siberia / northern Kazakhstan and Manych depression in Ciscaucasia, reaching Aral Sea and north Caspian lowlands in southern Russia in the west, bordered by main Caucasian Mountain Ridge from the south, southern border in Middle Asia roughly corresponds with Syrdarya valley and southern Ustyurt Plateau.
4. **Turan (TU):** formerly known as Turkestan, joins lowland arid areas of southern part of Middle Asia, including deserts of southernmost Kazakhstan, Uzbekistan, Turkmenistan, Tajikistan and northernmost Afghanistan, southwards from Syrdarya Valley, Aral Sea and southern Ustyurt, bordered by Caspian Sea in the west, Kopet Dagh Mts. and Hindukush Mts. in the south, bordered by Pamiro-Alai Mts. in the east.
5. Middle East (ME): arid areas of Iran, Afghanistan and western Pakistan eastwards from Zagros Mts., southwards from Elburz, Kopet Dagh and Hindukush Mts. and eastwards from Indus Valley.
6. **Asia Minor and Transcaucasia (MI):** Arid montane areas of Transcaucasia (Georgia, Armenia, Azerbaijan) and eastern Turkey (Asia Minor) including northern parts of Syria, Iraq and Iran.
7. **Near East and Arabia (AR):** lowland arid areas of Arabian Peninsula and Near East (Syria, Israel, Jordan, Iraq, westernmost Iran) bordered by Zagros Mts. in the east, and by Mediterranean Sea and Sinai in the west.

**References cited in the Supplementary Text Files:**

Ananjeva NB, Tuniev BS. 1992. Historical biogeography of the *Phrynocephalu*s species of the USSR. *Asiatic Herpetological Research* 4:76–98.

Bogdanov OP. 1965. *Ecology of Middle Asian reptiles.* Tashkent 258 p. [in Russian]

Chernov SA. 1949. *Reptiles of Tajikistan and genesis of modern herpetofauna of Middle Asia.* Doctoral thesis abstract, Leningrad, 9. [in Russian]

Chkhikvadze VM, Shammakov SM, Zerova GA. 1983. On the history of the formation of the fauna of Squamata of Middle Asia and Kazakhstan. *Izvestiya AN TurkmSSR, Biological series* 2:3–8. [in Russian]

Geptner VG. 1938. Zoogeographical features of the desert fauna of Turkestan and its origin. *Bulletin of Moscow Society of Nature Researchers, Biological series* 47(5-6):329–338. [in Russian]

Korovin EP. 1961. *The vegetation of Middle Asia and southern Kazakhstan. 1.* Tashkent, UzbekSSR Academy of Sciences 452. [in Russian]

Nikolsky AM. 1915. *Reptiles (Reptilia). Vol 1. Chelonia and Sauria. Fauna of Russia and adjacent countries.* Imp. Acad. Sci., Petrograd 534. [in Russian]

Rustamov AK, Szczerbak NN. 1985. The herpetogeographical separation of the region of Middle Asia. In: *Problemy Gerpetologii*. Leningrad: 181–182. [in Russian]

Sindaco R, Jeremcenko [Eremchenko] VK. 2008. *The Reptiles of the Western Palearctic. 1.* *Annotated checklist and distribution atlas of the turtles, crocodiles, amphisbaenian and lizards of Europe, North Africa, Middle East and Central Asia.* Edizioni Belvedere, Latina 579.
