# Supplementary figures and images for: Cenozoic aridization in Central Eurasia shaped diversification of toad-headed agamas (Phrynocephalus; Agamidae, Reptilia)
Source: PeerJ. 2018 Mar 19;6:e4543. doi: 10.7717/peerj.4543 (PMC5863718; doi:10.7717/peerj.4543)

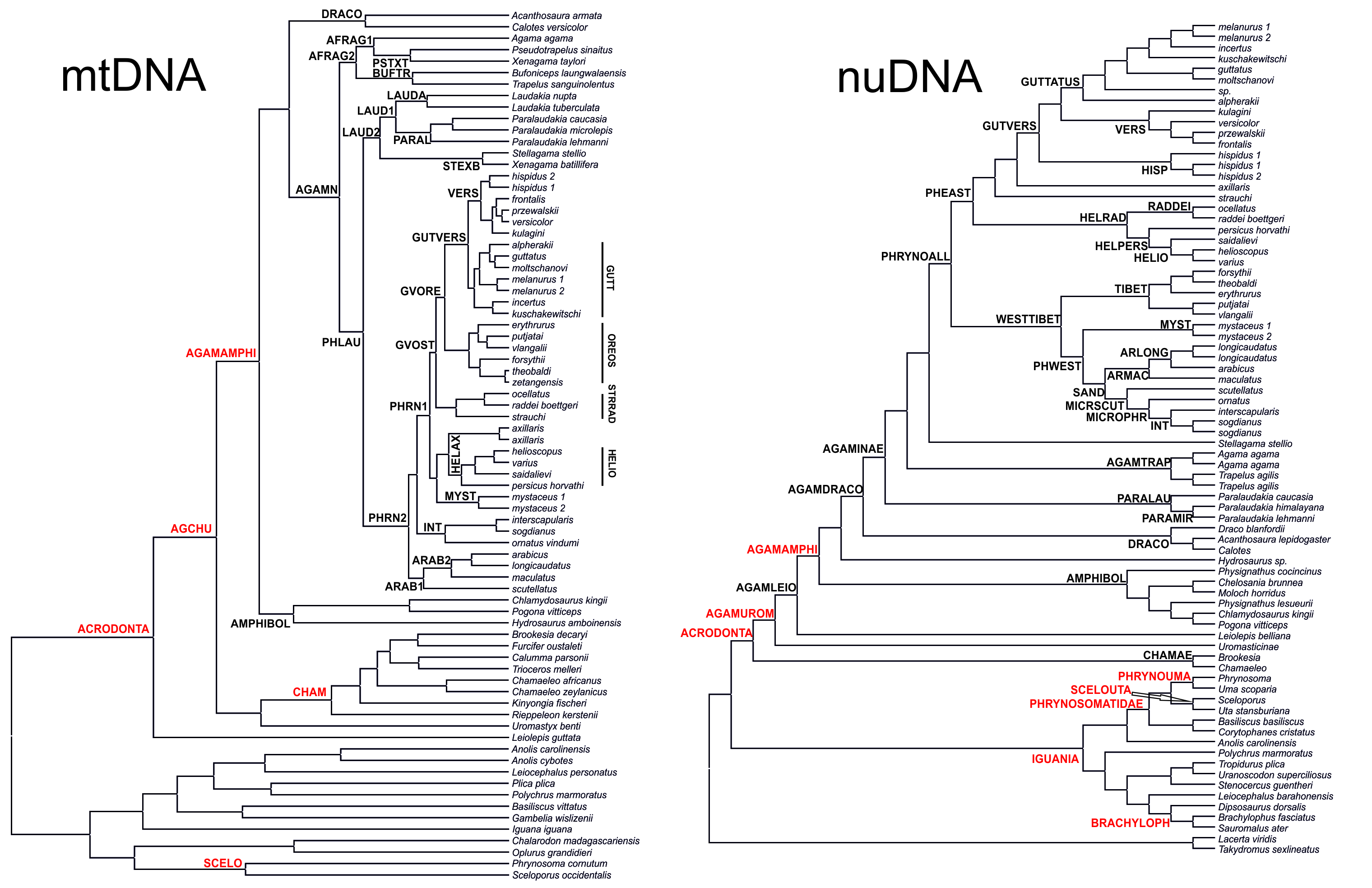

Supplement: Supplemental Information 4 — See Table S5 for calibrations and Table S10 for divergence time estimates. [file peerj-06-4543-s004.png]

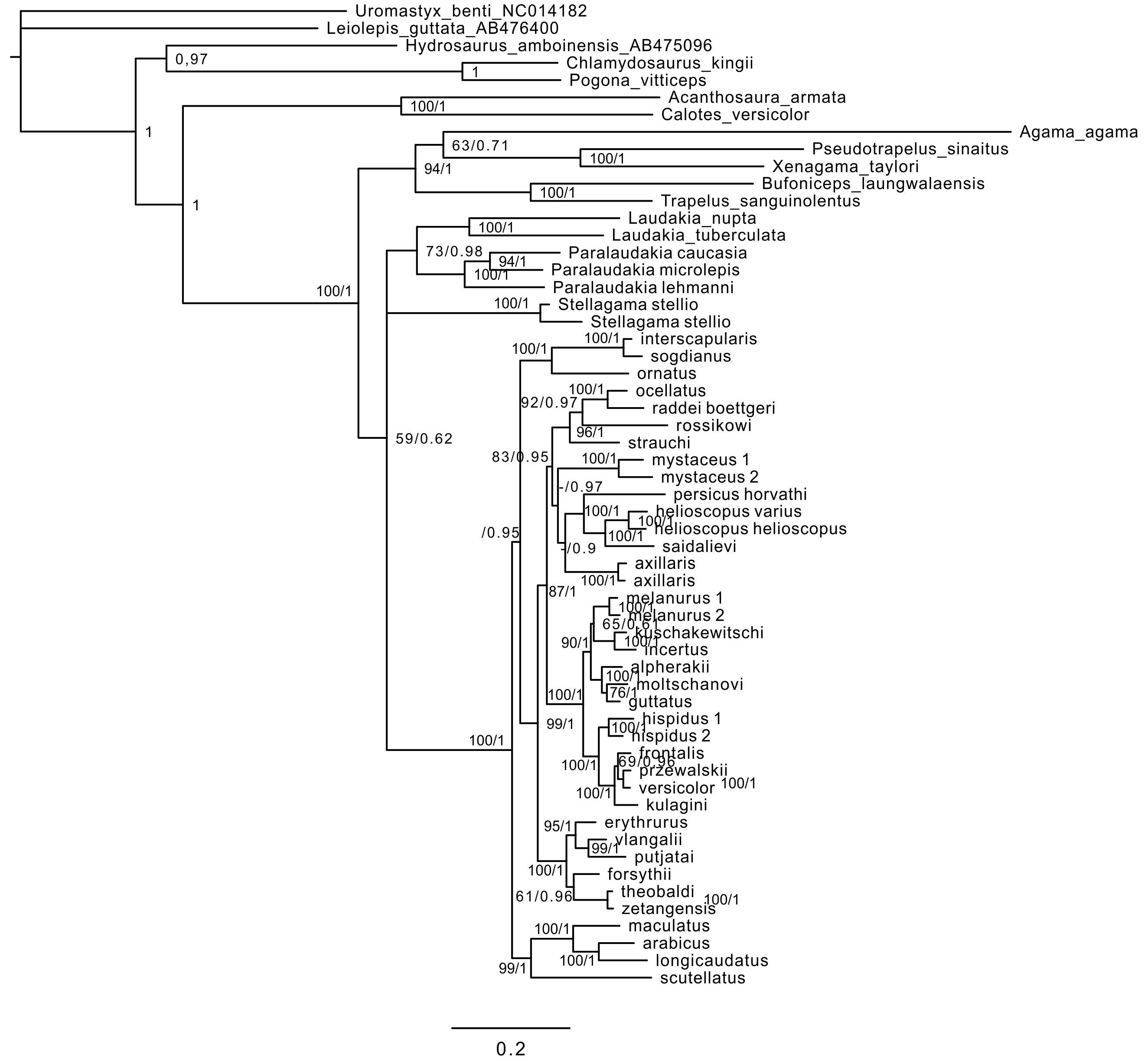

Supplement: Supplemental Information 5 — ML BSP/BI BPP values are given for resolved nodes only. [file peerj-06-4543-s005.png]

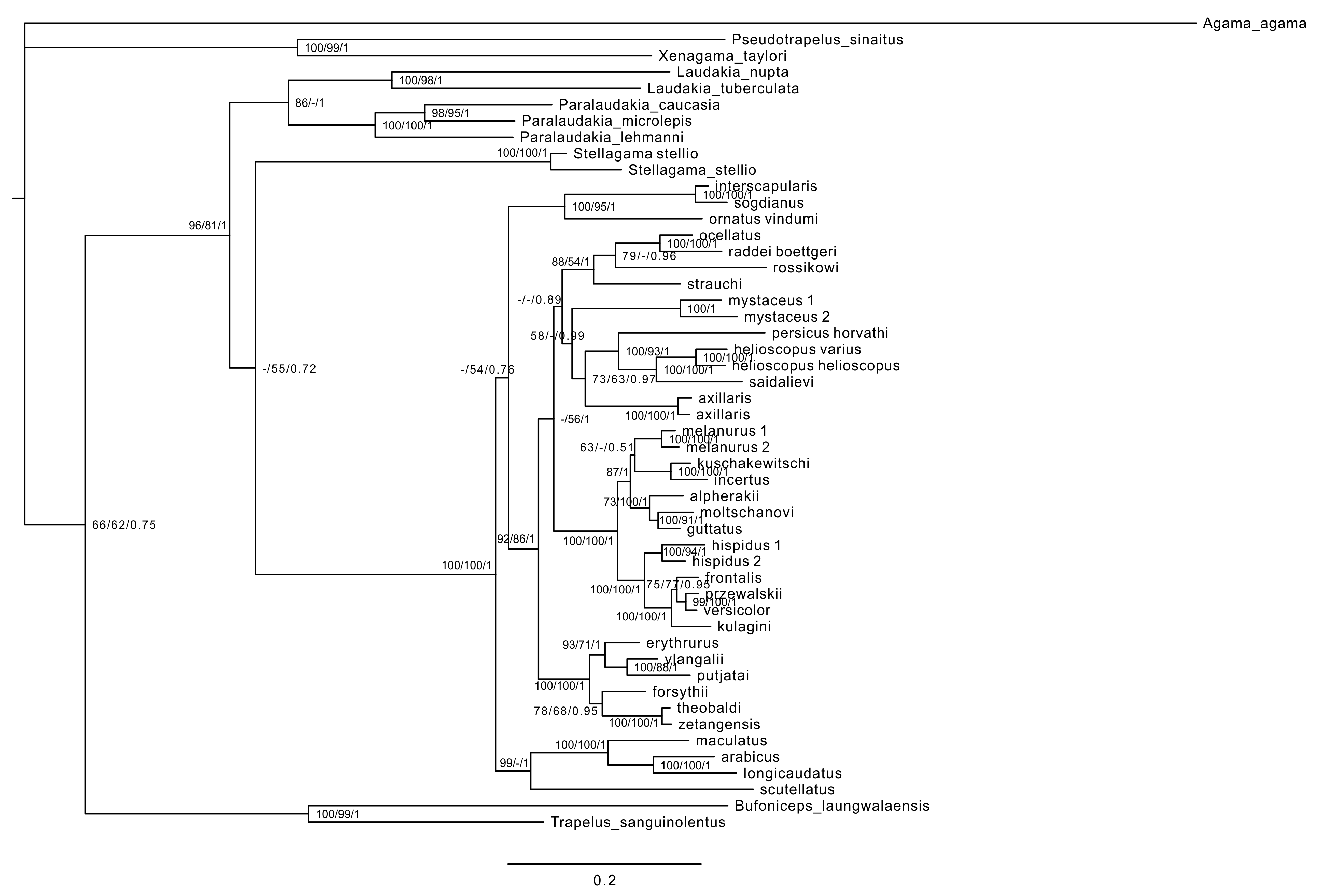

Supplement: Supplemental Information 6 — ML BSP/BI BPP values are given for resolved nodes only. [file peerj-06-4543-s006.png]

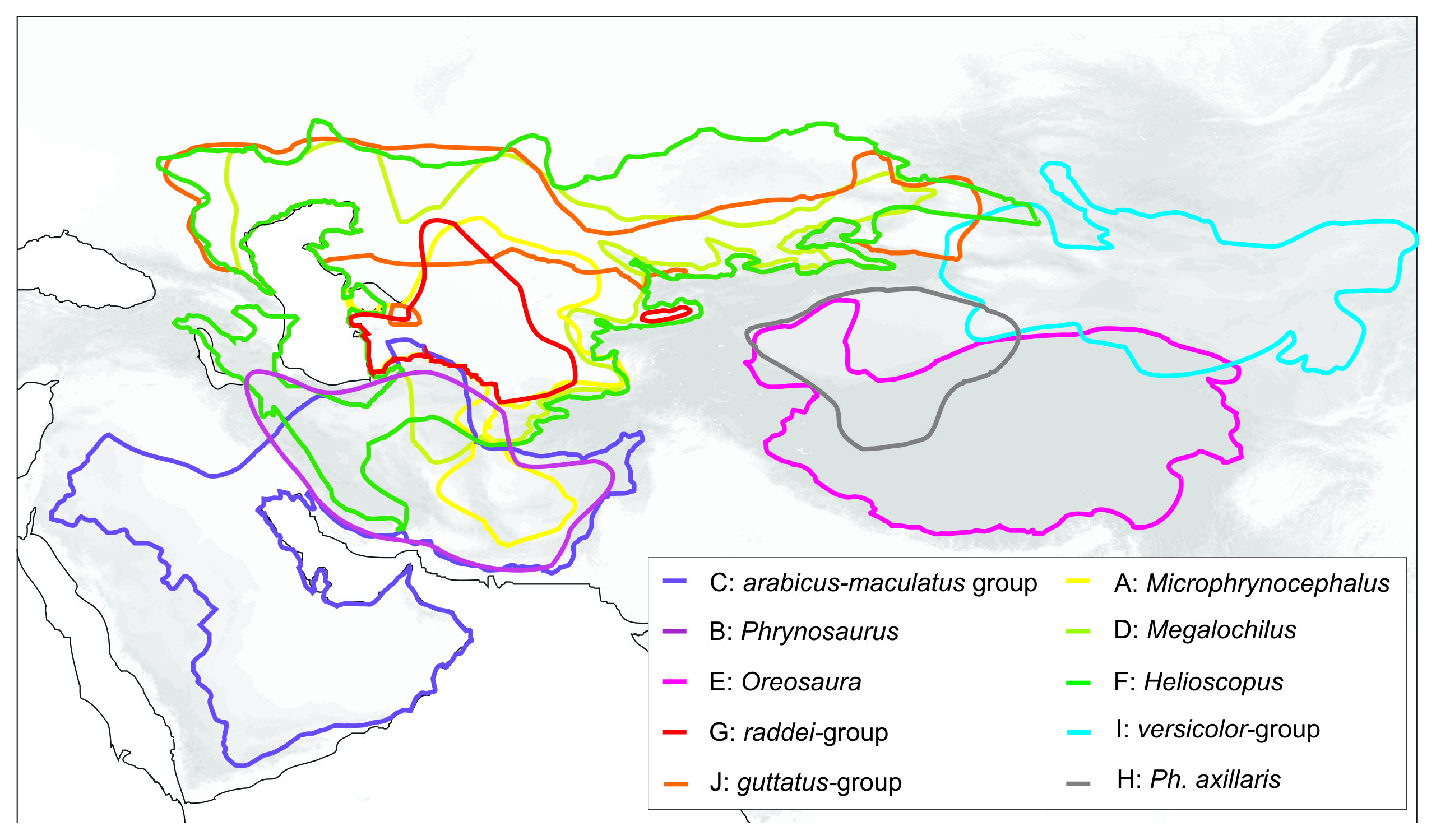

Supplement: Supplemental Information 7 — Color marking of species groups corresponds to Figs. 2 and 3. [file peerj-06-4543-s007.png]

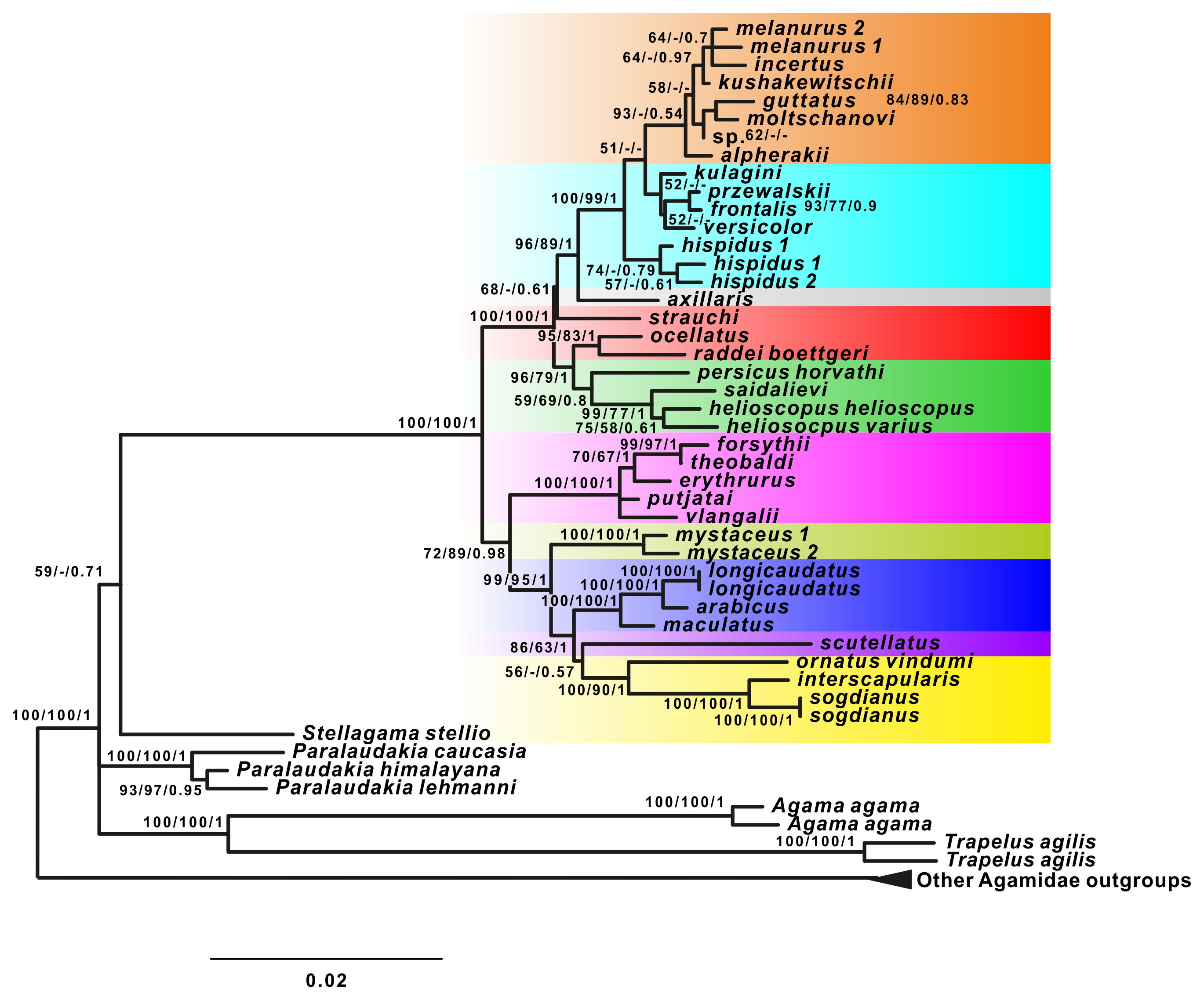

Supplement: Supplemental Information 8 — ML BSP/BI BPP values are given for resolved nodes only. [file peerj-06-4543-s008.png]

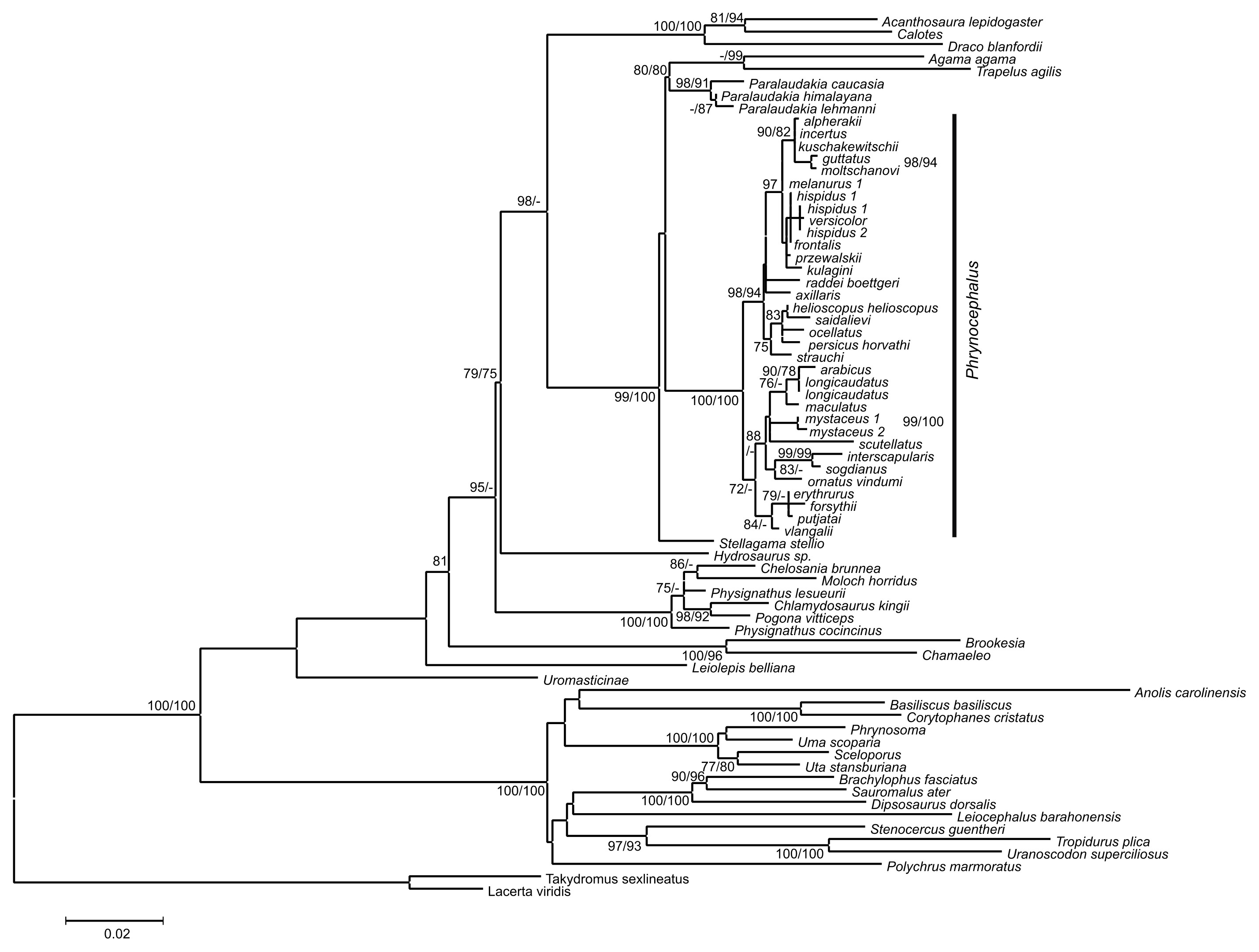

Supplement: Supplemental Information 9 — ML BSP/MP BSP values are given for strongly supported nodes. [file peerj-06-4543-s009.png]

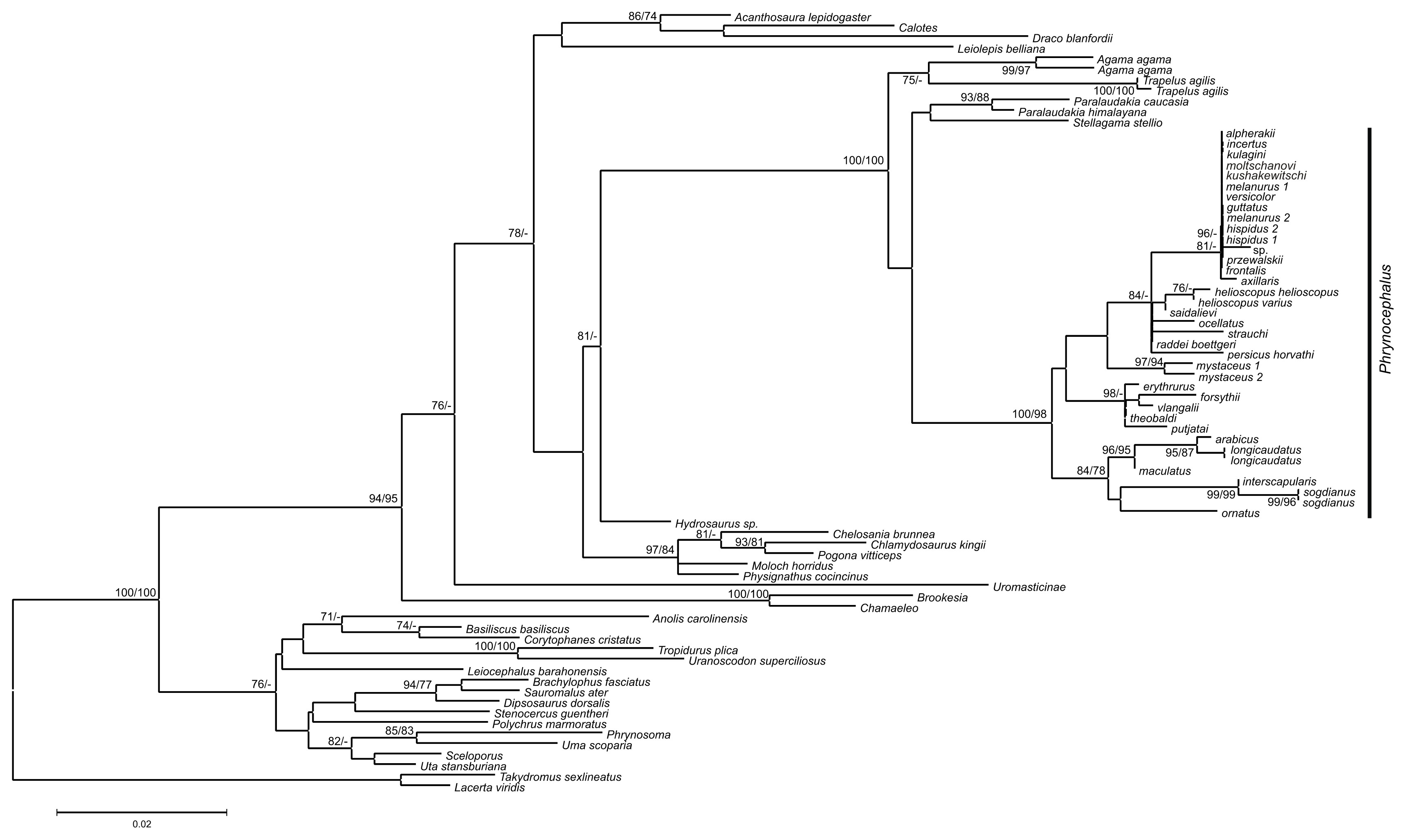

Supplement: Supplemental Information 10 — ML BSP/MP BSP values are given for strongly supported nodes. [file peerj-06-4543-s010.png]

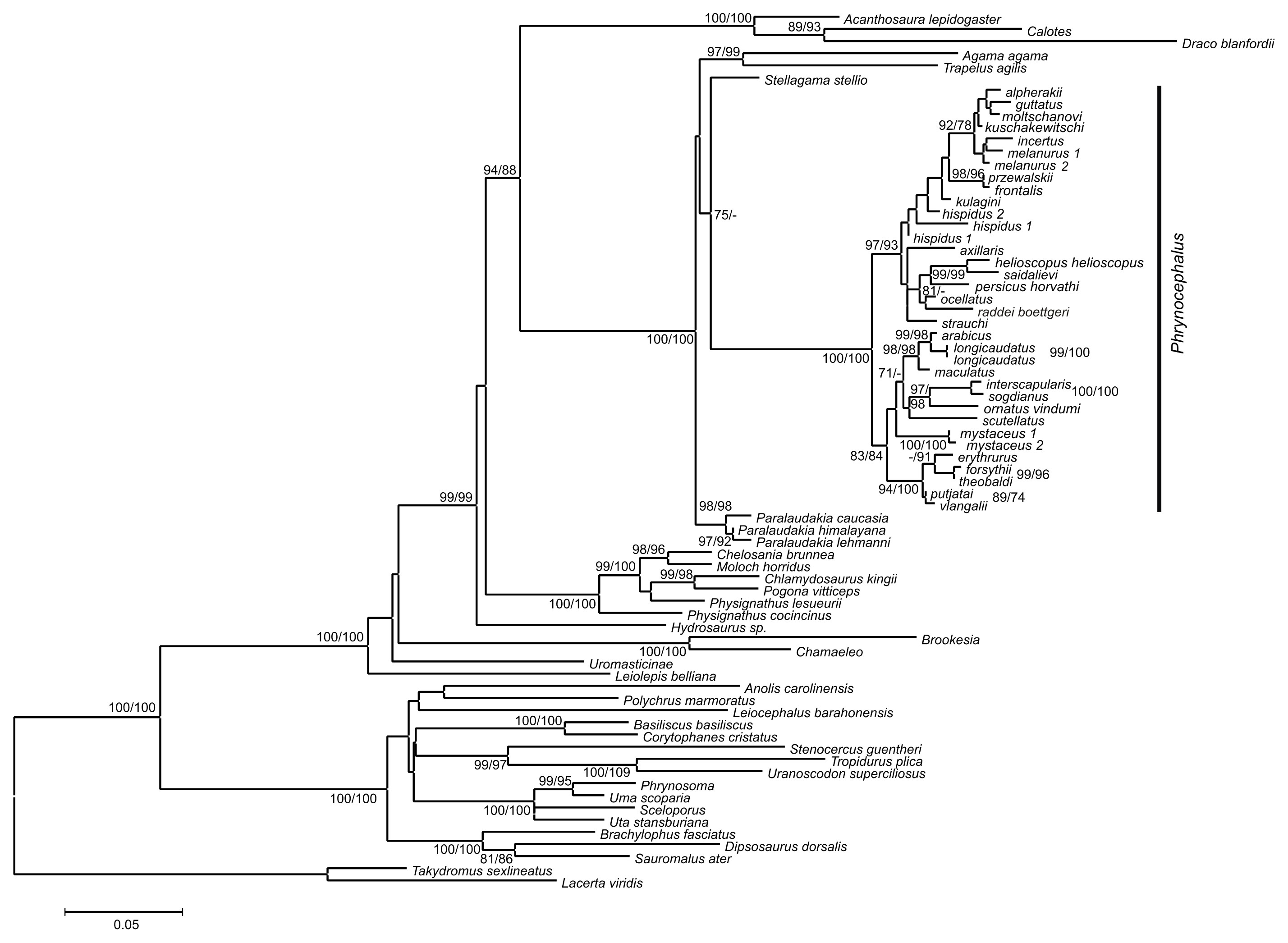

Supplement: Supplemental Information 11 — ML BSP/MP BSP values are given for strongly supported nodes. [file peerj-06-4543-s011.png]

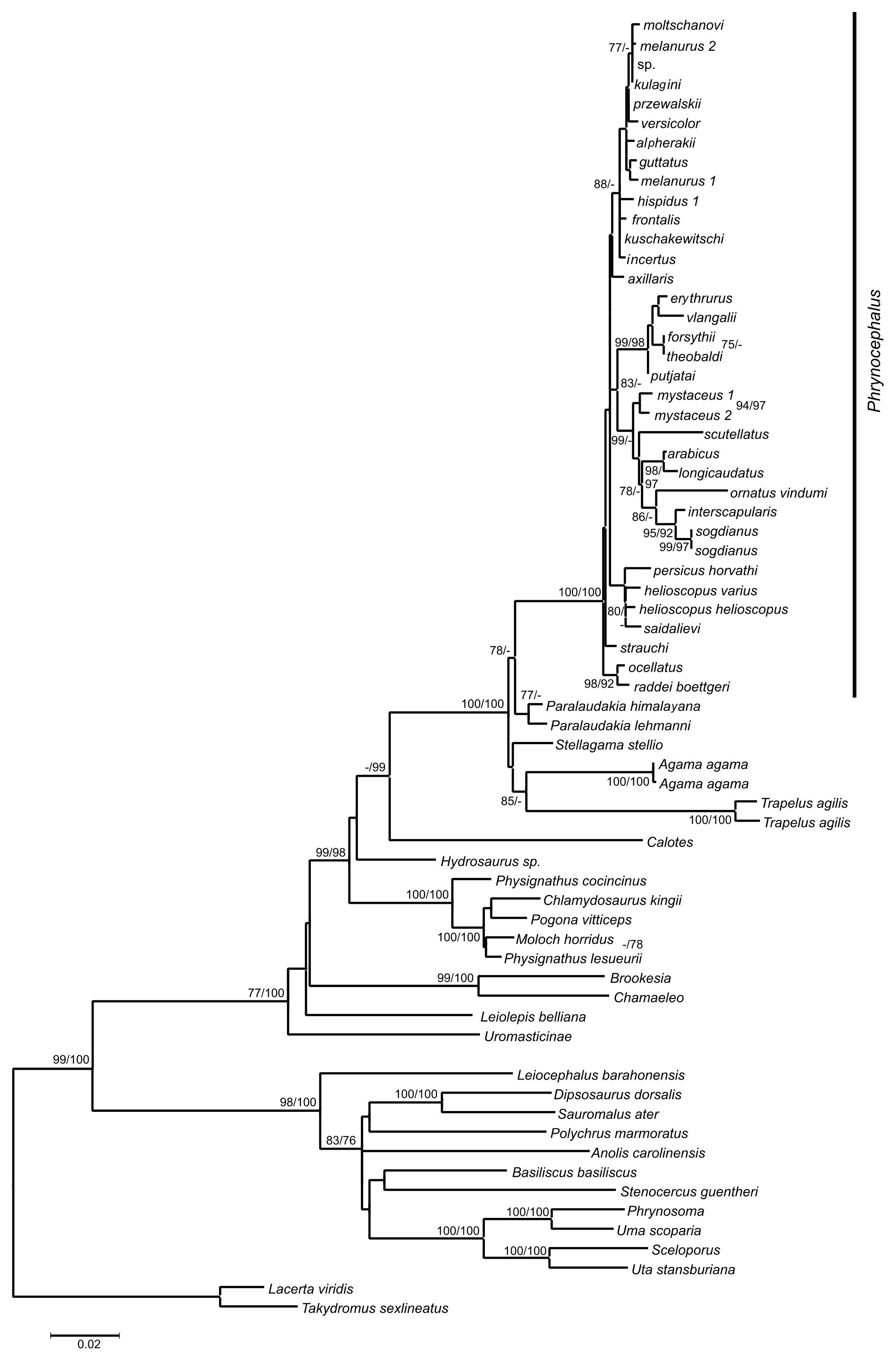

Supplement: Supplemental Information 12 — ML BSP/MP BSP values are given for strongly supported nodes. [file peerj-06-4543-s012.png]

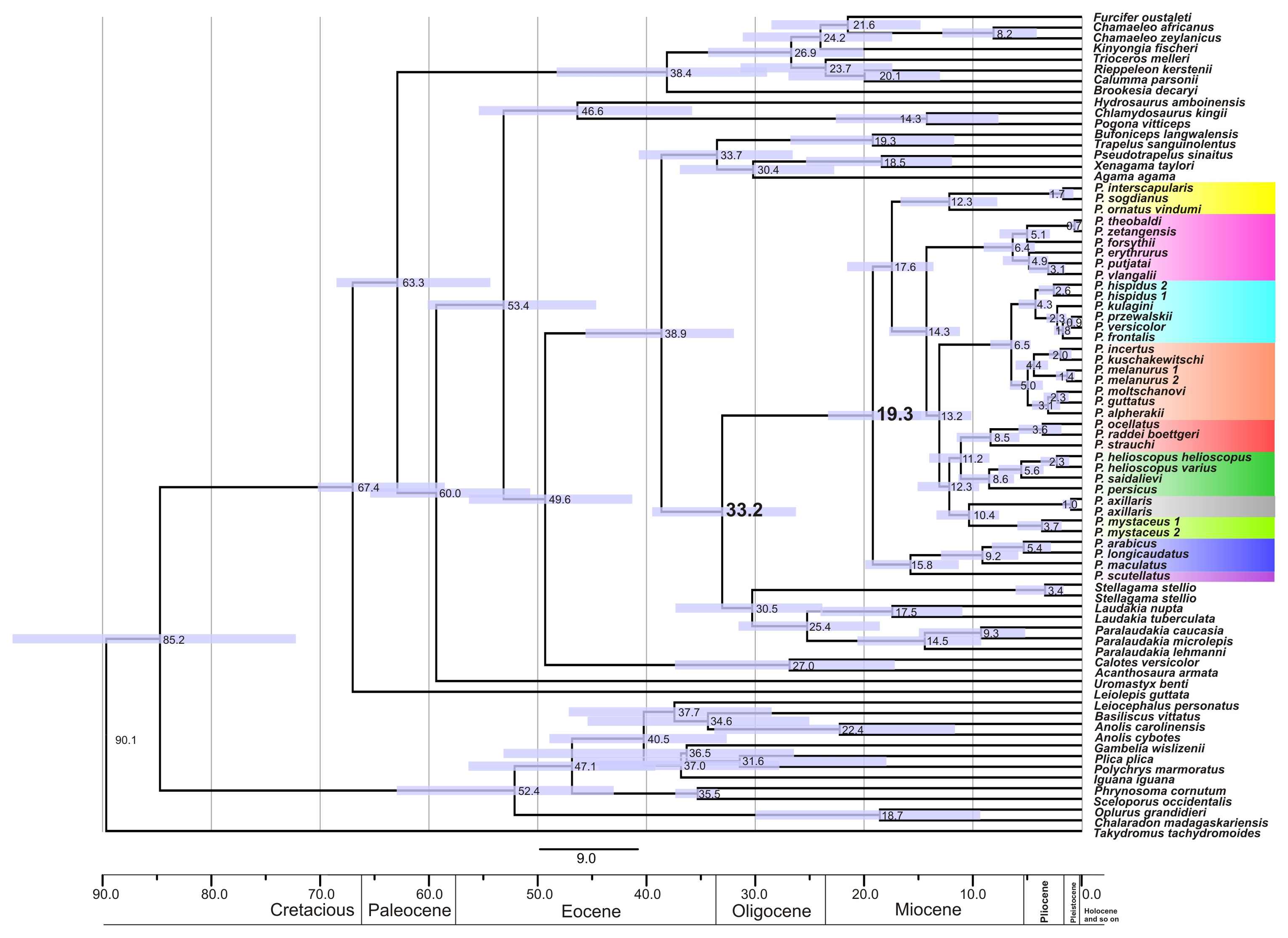

Supplement: Supplemental Information 13 — Node values correspond to estimated divergence times (in Ma). Grey-bars correspond to 95%-confidence intervals. Color marking of species groups corresponds to Figs. 2 and 3. [file peerj-06-4543-s013.png]

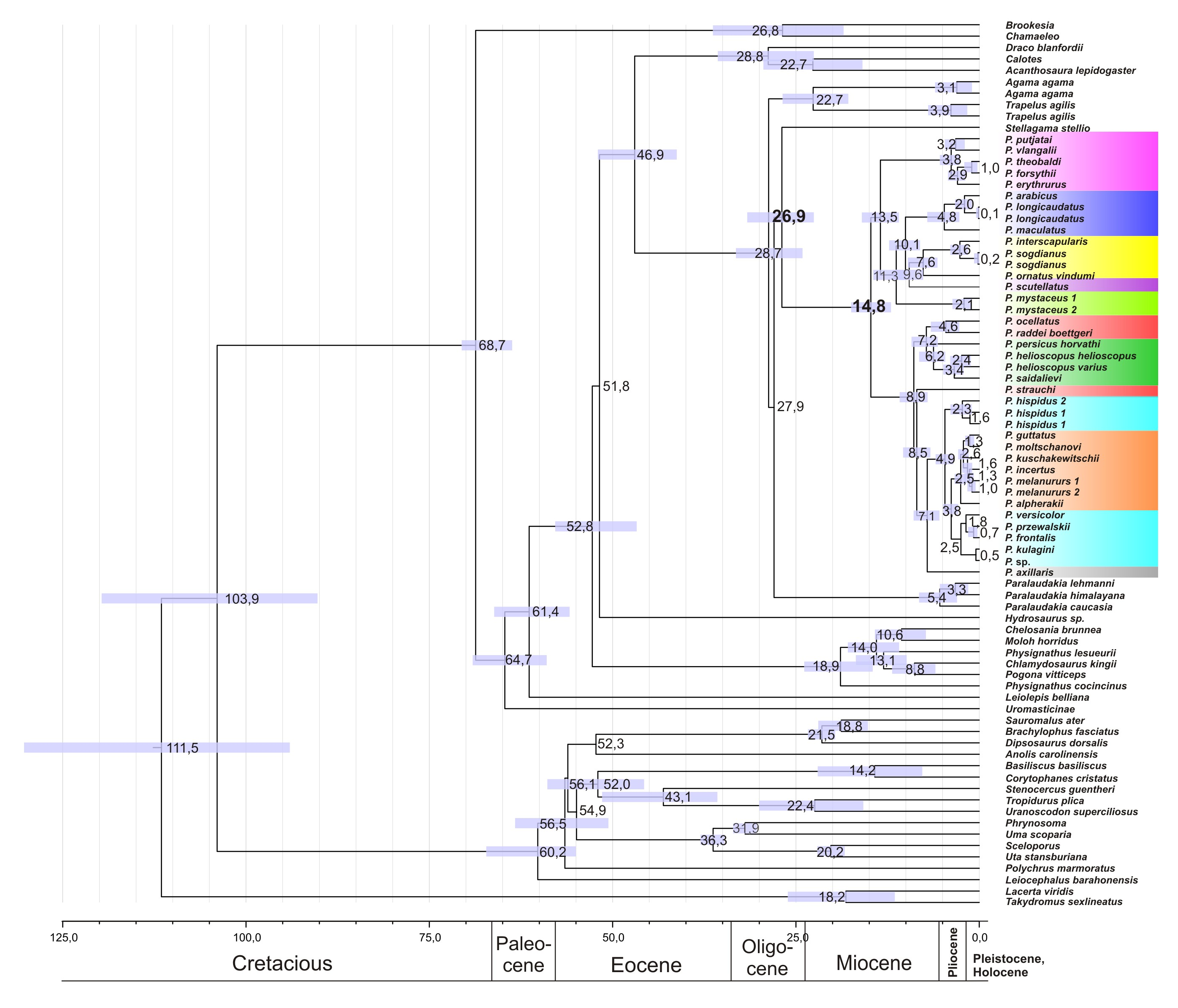

Supplement: Supplemental Information 14 — Node values correspond to estimated divergence times (in Ma). Grey-bar corresponds to 95%-credibility interval. Color marking of species groups corresponds to Figs. 2 and 3. [file peerj-06-4543-s014.png]

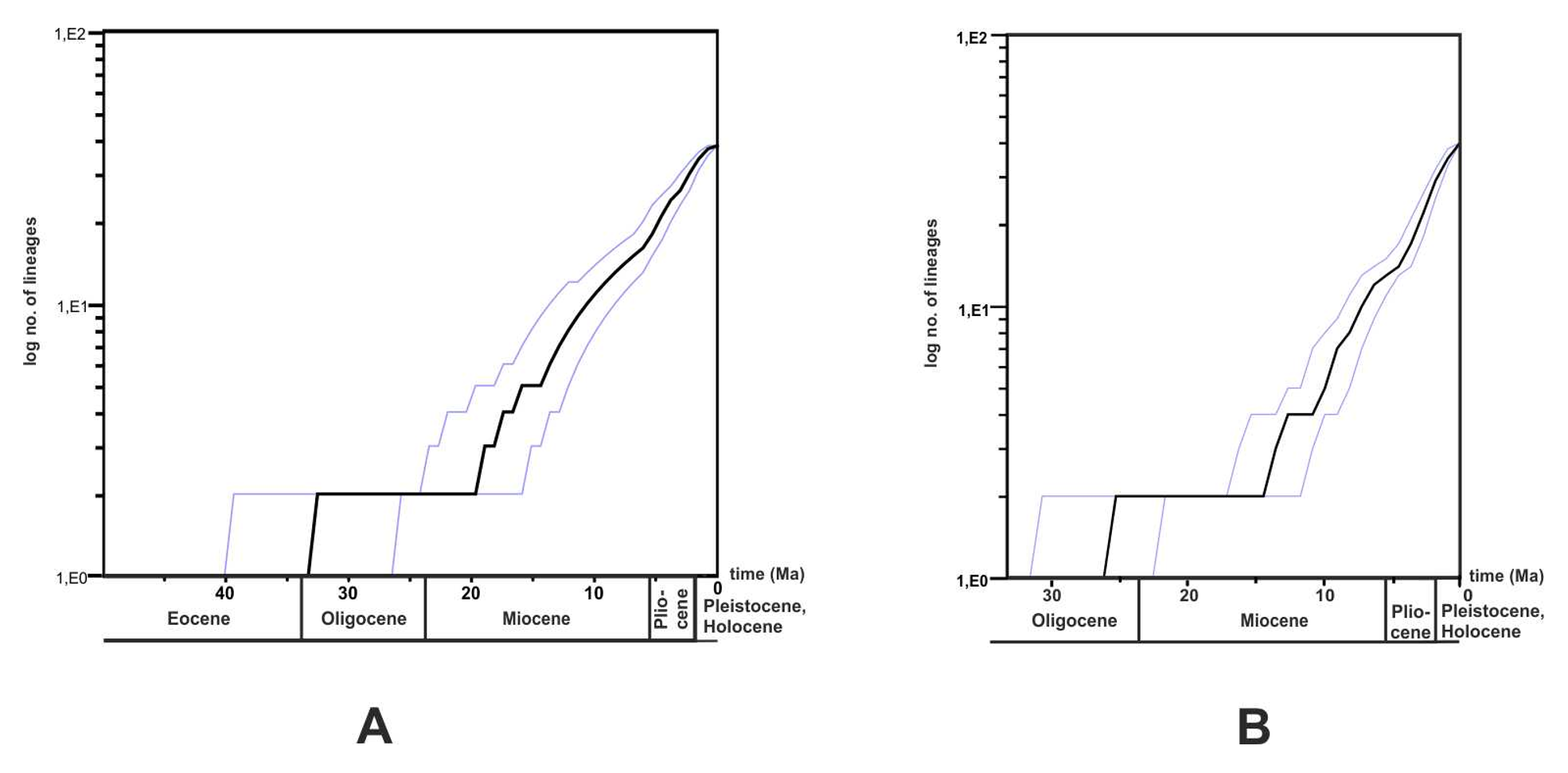

Supplement: Supplemental Information 15 [file peerj-06-4543-s015.png]
