# Supplementary material for: Cenozoic aridization in Central Eurasia shaped diversification of toad-headed agamas (Phrynocephalus; Agamidae, Reptilia)
Source: PeerJ. 2018 Mar 19;6:e4543. doi: 10.7717/peerj.4543 (PMC5863718; doi:10.7717/peerj.4543)
Supplement: Supplemental Information 16 — ID/source–museum voucher/isolate ID; Locality–geographic locality of origin. No exact locality information is available for specimens obtained via pet trade. [file peerj-06-4543-s016.docx]

| **Species** | **ID/source** | **Locality** |
| --- | --- | --- |
| *Agama agama* | Aag | *Pet trade* |
| *Laudakia nupta* | ZMMU R-12711 | Iran, Kermanshah, Bisotun, N34.37, E47.43 |
| *Paralaudakia caucasia* | ZMMU R-12465 | Azerbaijan, Zuvand, Kyalvyaz, N41.52, E46.25 |
| *Paralaudakia caucasia* | ZMMU R-13762 | Armenia |
| *Paralaudakia himalayana* | ZMMU R-13407 | Tajikistan, Badakhshan |
| *Paralaudakia lehmanni* | ZMMU R-12248 | Uzbekistan, Navoi dist., Nuratau, N40.61, E66.58 |
| *Paralaudakia microlepis* | ZMMU R-12207 | Iran, Khorasan, Birjant, Sedekh |
| *Phrynocephalus alpherakii* | ZMMU R-12667 | Kazakhstan, Ily river valley, Tashkarasu, N43.73, E79.47 |
| *P. alpherakii* | ZMMU R-12670 | Kazakhstan, Ily river valley, 25 km E from Kokpek, N43.45, E78.90 |
| *P. alpherakii* | ZMMU R-12811 | Kazakhstan, right bank of Ily river, near Aktau mts., N44.083, E79.47 |
| *P. arabicus* | ZMMU R-12713 | Iran, Khujestan |
| *P. axillaris* | ZMMU R-13087 | China, Xinjiang Uygur Zizhiqu, N40.87, E87.90 |
| *P. axillaris* | ZMMU R-12302 | China, Xizang, Autonomous Region, environs of Rutog, N33.68, E80.31 |
| *P. erythrurus* | ZMMU R-12303 | China, Qinghai, environs of Duokake, KunLun Shan (KunLun mt.), N36.61, E92.33 |
| *P. forsythii* | ZMMU R-12301 | China, Xinjiang Uygur AR, env. of Pelguzo lake, N28.66, E85.88 |
| *P. frontalis* | Pprz | China, *Pet trade* |
| *P. guttatus* | ZMMU R-12174 | Russia, Astrakhan dist., Dosang |
| *P. guttatus* | Pgu1 | Russia, Astrakhan dist., Dosang |
| *P. helioscopus helioscopus* | ZMMU R-13250-1,2 | Uzbekistan, env. of Kyzylkum Botanical Station |
| *P. helioscopus helioscopus* | ZMMU R-12525 | Kazakhstan, Mangistauss area, Ustyurt Nature reserve, N42.88, E53.08 |
| *P. helioscopus varius* | ZMMU R-13089 | China, Xinjiang Uygur Autonomous Region, Toli env., N45.93, E83.65 |
| *P. helioscopus varius* | ZMMU R-12524 | Kazakhstan, near Zhalanashkol lake, N45.57, E82.21 |
| *P. hispidus* | KIZ 014303 | China, Gansu |
| *P. hispidus* | ZMMU R-12827 | Mongolia, Bayanhongor, Transaltay Gobi, 35 km N from fruit farm in Echiyn-Gol tract, 15 km S from Nogon-Tzav tract, N43.48, E99.12 |
| *P. hispidus* | ZMMU R-12828 | Mongolia, Bayanhongor, Transaltay Gobi, 1 km S from fruit farm in Echiyn-Gol tract, E43.23, E99 |
| *P. hispidus* | ZMMU R-12837 | Mongolia, Umnegov, Transaltay Gobi, 90 km E from fruit farm in Echiyn-Gol tract, Sertengiyn-Bosgo tract on the W from Tost-Uul mountains, N43.22, E100.07 |
| *P. incertus* | ZMMU R-13088 | Kazakhstan, N46.68, E75.45 |
| *P. incertus* | ZMMU R-12769 | Kazakhstan, right bank of Kapchagay reservoir, 43.88, E77.69 |
| *P. incertus* | ZMMU R-12673 | Kazakhstan, Ily river valley, N from Pidzhim, N44.15, E80.22 |
| *P. interscapularis* | ZMMU R-12268 | Uzbekistan, Chukurkak village env., N42.65, E61.55 |
| *P. interscapularis* | ZMMU R-13358 | Uzbekistan, Nurata dist., N40.82, E66.63 |
| *P. kulagini* | ZMMU R-12939-1,2 | Russia, Tuva, TereKhol lake env., Tsugeer-Els sands, N50.65, E93.25 |
| *P. kuschakewitschi* | ZMMU R-12516 | Kazakhstan, Zhamankum sands |
| *P. kuschakewitschi* | ZMMU R-12173 | E Kazakhstan, environs of Shagantoghai and Emel confluence |
| *P. longicaudatus* | ZMMU R-13899-1,2 | Oman,40 km NE Filim |
| *P. maculatus* | ZMMU R-13029 | Iran, Esfahan, Chupanan, N33.52, E54.25 |
| *P. melanurus* 1 | ZMMU R-12767 | Kazakhstan, Karatal river, N45.67, E77.25 |
| *P. melanurus* 1 | ZMMU R-12332 | Kazakhstan, env. of Bukhtarma reservoir, N48.84, E83.43 |
| *P. melanurus* 2 | ZMMU R-12327 | Kazakhstan, Alakol valley, between Alakol and Zhalanashkol lakes, 55 km S from Karabulak, N45.64, E82.17 |
| *P. melanurus* 2 | Pfr | China, *Pet trade* |
| *P. moltschanovi* | ZMMU R-12942 | Uzbekistan, Beltau, E from Takhtakupyr, N43.33,E60.92 |
| *P. mystaceus* 1 | Pmy2 | Russia, Astrakhan dist., Dosang |
| *P. mystaceus* 1 | ZMMU R-12261 | Uzbekistan, Navoi dist., Yamankum desert, 3 - 4 W from Aktakyr, N41.74, E64.02 |
| *P. mystaceus* 2 | ZMMU R-13009 | Iran, Khorasan, Boshruye, N33.90, E57.5 |
| *P. ocellatus* (= *P. reticulatus*) | ZMMU R-12272 | Uzbekistan, W border of Minbulak hollow, 25 km SW Minbulak, N42.12, E62.74 |
| *P. ocellatus* (= *P. reticulatus*) | ZMMU R-13359 | Uzbekistan, env. of Kyzylkum Botanical Station, N40.83, E63.73 |
| *P. ornatus vindumi* | ZMMU R-13026 | Iran, Khorasan, Gonobad, N34.76, E58.78 |
| *P. ornatus vindumi* | ZMMU R-13027 | Iran, Khorasan, Gonobad |
| *P. persicus* | ZMMU R-12466 | Azerbaijan, Nakhichevan, N39.22, E45.42 |
| *P. persicus* | ZMMU R-13243-1,2,3 | Armenia, Gorovan sands, N39.92, E44.73 |
| *P. persicus* | ZMMU R-12322 | Armenia, 15 km NE Armavir, N40.15, E44.03 |
| *P. przewalskii* | KIZ 014306 | China, Ningxia |
| *P. przewalskii* | ZMMU R-8900 | China, Gansu, Lanzhou, Lanzhou, university territory, N36.03, E103.41 |
| *P. putjatai* | KIZ 020238 | China, Qinghai, env. of. Qinghai lake |
| *P. putjatai* | ZMMU R-12308 | China, Qinпhai, NE of Kukunor lake |
| *P. raddei boettgeri* | ZMMU R-14984 | Uzbekistan, Surkhandarya dist. |
| *P. raddei* | IZIP998 | Uzbekistan, Surkhandarya dist., Termez |
| *P. rossikowi* | ZMMU R-12795 | Turkmenistan, Chardzhou, Dargan-Ata, Lebap, N41.05, E61.87 |
| *P. saidalievi* | ZMMU R-12802-1,2 | Uzbekistan, Fergana Valley, near Kokand, N40.52, E70.93 |
| *P. scutellatus* | ZMMU R-13020 | Iran, Esfahan, Chupanan, N33.52, E54.25 |
| *P. sogdianus* | ZMMU R-13396-1,2 | S Tajikistan, Kurjalakum sands, N37.31, E68.29 |
| *P. sogdianus* | ZMMU R-12800 | Uzbekistan, Surkhandarya dist., 10-15 km from Dzharkurgan to Termez, N37.33, E67.37 |
| *P.* sp. | ZMMU R-12309 | China, Inner Mongolia, Ordos desert |
| *P. strauchi* | ZMMU R-13247 | Uzbekistan, Fergana Valley, between Andijan and Kokand, N40.80, E71.24 |
| *P. strauchi* | ZMMU R-11386 | Uzbekistan, Fergana Valley, 40 km from Kokand to Andizhan, N40.69, E71.90 |
| *P. theobaldi theobaldi* | ZMMU R-10816 | India, Dzhammu and Kashmir, Ladak, Teokar valley, N33.01, E77.99 |
| *P. theobaldi zetangensis* | ZMMU R-12138 | India, Dzhammu and Kashmir, Ladak |
| *P. versicolor* | ZMMU R-12178 | Mongolia, South-Goby Aymak, 35 km NW Dalanzadgad, N43.78, E107.01 |
| *P. versicolor* | ZMMU R-12834 | Mongolia, Bayan-Khongor aymak, S Shangai, 25 - 30 km E from Zhinst-somon, N45.55, E10.27 |
| *P. vlangalii* | KIZ 020062 | China, Qinghai, Xinghai |
| *Stellagama stellio* | ZMMU R-11324 | Israel, Rehovat, N31.88, E34.79 |
| *Stellagama stellio* | ZMMU R-13375 | Greece, Kos Island |
| *Trapelus agilis* | Tag | *Pet trade* |
| *Trapelus sanguinolentus* | ZMMU R-12732 | Iran, Mazendaran, 40 km NNW Gonbad-e-Kabus, N37.48, E54.95 |
| *Trapelus sanguinolentus* | ZMMU R-12709 | Iran, Khorasan, near Garm Ab, N37.90, E56.43 |
