# Supplementary material for: Cenozoic aridization in Central Eurasia shaped diversification of toad-headed agamas (Phrynocephalus; Agamidae, Reptilia)
Source: PeerJ. 2018 Mar 19;6:e4543. doi: 10.7717/peerj.4543 (PMC5863718; doi:10.7717/peerj.4543)
Supplement: Supplemental Information 17 — Specimen ID/source: Museum ID or GB–sequences downloaded from GenBank. [file peerj-06-4543-s017.docx]

| **Species** | **Specimen ID/source** | ***COI*** | ***cytb*** | ***ND2*** | ***ND4*** | ***NKTR*** | ***AKAP9*** | ***BDNF*** | ***RAG-1*** |
| --- | --- | --- | --- | --- | --- | --- | --- | --- | --- |
| *Acanthosaura lepidogaster* | GB | - | - | - | - | JF805815 | JF805815 | JF805815 | - |
| *Acanthosaura armata* | GB | NC014175 | NC014175 | NC014175 | NC014175 | - | - | - | - |
| *Agama agama* | Aag | - | - | - | - | - | - | KJ363400 | KJ363479 |
| *Agama agama* | GB | NC014175 | AB263940 | AF128504 | AF443225 | JF805816 | JF805816 | JF805816 | JF805816 |
| *Anolis carolinensis* | GB | NC010972 | NC010972 | NC010972 | NC010972 | JF805835 | JF805835 | JF805835 | JF805835 |
| *Anolis cybotes* | GB | AB218960 | AB218960 | AB218960 | AB218960 | - | - | - | - |
| *Basiliscus basiliscus* | GB | - | - | - | - | JF805836 | JF805836 | JF805836 | JF805836 |
| *Basiliscus vittatus* | GB | NC012829 | NC012829 | NC012829 | NC012829 | - | - | - | - |
| *Brachylophus fasciatus* | GB | - | - | - | - | JF805837 | JF805837 | JF805837 | - |
| *Brookesia* | GB | NC014174 | NC014174 | NC014174 | NC014174 | JF805817 | JF805817 | JF805817 | AY662577 |
| *Bufoniceps laungwalaensis* | GB | DQ008214 | DQ008214 | DQ008214 | DQ008214 | - | - | - | - |
| *Calotes* | GB | - | - | - | - | JF805818 | JF805818 | JF805818 | AY662584 |
| *Calotes versicolor* | GB | NC009683 | NC009683 | NC009683 | NC009683 | - | - | - | - |
| *Calumma parsonii* | GB | AB474915 | AB474915 | AB474915 | AB474915 | - | - | - | - |
| *Chalarodon madagascariensis* | GB | NC012836 | NC012836 | NC012836 | NC012836 | - | - | - | - |
| *Chamaeleo* | GB | - | - | - | - | JF805819 | JF805819 | JF805819 | AY662578 |
| *Chamaeleo africanus* | GB | NC012422 | NC012422 | NC012422 | NC012422 | - | - | - | - |
| *Chamaeleo zeylanicus* | GB | EF222191 | EF222191 | EF222191 | EF222191 | - | - | - | - |
| *Chelosania brunnea* | GB | - | - | - | - | JF805820 | JF805820 | JF805820 | - |
| *Chlamydosaurus kingii* | GB | NC009421 | NC009421 | NC009421 | NC009421 | JF805821 | JF805821 | JF805821 | JF805821 |
| *Corytophanes cristatus* | GB | - | - | - | - | JF805839 | JF805839 | JF805839 | - |
| *Dipsosaurus dorsalis* | GB | - | - | - | - | JF805841 | JF805841 | JF805841 | JF805841 |
| *Draco blanfordii* | GB | - | - | - | - | JF805824 | JF805824 | JF805824 | - |
| *Furcifer oustaleti* | GB | AB185326 | AB185326 | AB185326 | AB185326 | - | - | - | - |
| *Gambelia wislizenii* | GB | NC012831 | NC012831 | NC012831 | NC012831 | - | - | - | - |
| *Hydrosaurus* | GB | AB475096 | AB475096 | AB475096 | AB475096 | JF805825 | JF805825 | JF805825 | JF805825 |
| *Iguana iguana* | GB | NC002793 | NC002793 | NC002793 | NC002793 | - | - | - | - |
| *Kinyongia fischeri* | GB | EF222188 | EF222188 | EF222188 | EF222188 | - | - | - | - |
| *Lacerta* | GB | - | - | - | - | JF805800 | JF805800 | JF805800 | EF632222 |
| *Laudakia nupta* | ZMMU R-12711, GB | HM915020 | - | AF128513 | KF691667 | - | - | - | - |
| *Laudakia tuberculata* | GB | - | - | AF128514 | - | - | - | - | - |
| *Leiocephalus* | GB | NC012834 | NC012834 | NC012834 | NC012834 | JF805844 | JF805844 | JF805844 | AY662598 |
| *Leiolepis* | GB | AB476400 | AB476400 | AB476400 | AB476400 | JF805827 | JF805827 | JF805827 | JF805827 |
| *Moloch horridus* | GB | - | - | - | - | JF805828 | JF805828 | JF805828 | JF805828 |
| *Oplurus grandidieri* | GB | NC012827 | NC012827 | NC012827 | NC012827 | - | - | - | - |
| *Paralaudakia caucasia* | ZMMU R-13762, ZMMU R-12465, GB | KF691701 | KF691617 | AF028687 | HQ919083 | KJ363440 | KJ363514 | DQ340732 | - |
| *Paralaudakia himalayana* | ZMMU R-13407 | - | - | - | - | KJ363441 | KJ363515 | KJ363402 | KJ363481 |
| *Paralaudakia lehmanni* | ZMMU R-12248, GB | KF691702 | KF691618 | KF691670 | AF028677 | KJ363442 | KJ363516 | - | KJ363482 |
| *Paralaudakia microlepis* | ZMMU R-12207 | KF691703 | KF691647 | – | KF691699 | - | - | - | - |
| *P. alpherakii* | ZMMU R-12667, ZMMU R-12670, ZMMU R-12811, GB | KF691729 | KF691639 | GQ242221 | KF691692 | KJ363456 | KJ363528 | KJ363417 | KJ363494 |
| *P. arabicus* | ZMMU R-12713 | KF691708 | – | KF691675 | KF691651 | KJ363472 | KJ363545 | KJ363434 | KJ363507 |
| *P. axillaris* | ZMMU R-12302, ZMMU R-13087, GB | KF691724 | KF691635 | - | - | KJ363449 | KJ363522 | KJ363410 | KC551397 |
| *P. axillaris* | GB | - | - | AY396570 | AY054012 | - | - | - | - |
| *P. erythrurus* | ZMMU R-12303, GB | KF691718 | KF691632 | GQ141231 | KF691684 | KJ363446 | KJ363519 | KJ363406 | KJ363485 |
| *P. forsythii* | ZMMU R-12301, GB | KF691720 | KF691633 | AY054017 | KF691661 | KJ363445 | KJ363518 | KJ363405 | KJ363484 |
| *P. frontalis* | ZMMU R-12309, GB | KF691695 | AY053909 | AY396592 | KF691733 | - | - | KJ363409 | - |
| *P. frontalis* | Pprz | - | - | - | - | KJ363462 | KJ363534 | KJ363422 | KJ363498 |
| *P. guttatus* | Pgu1, ZMMU R-12174, GB | KF691731 | KF691640 | GQ242203 | KF691693 | KJ363453 | KJ363525 | KJ363414 | KJ363491 |
| *P. helioscopus* | ZMMU R-13250-1,2, ZMMU R-12525 | KF691716 | KF691630 | KF691658 | KF691683 | KJ363465 | KJ363538 | KJ363426 | KJ363502 |
| *P. hispidus* | KIZ 014303 | KF691738 | KF691646 | – | – | KJ363457 | KJ363529 | KJ363418 | - |
| *P. hispidus* | ZMMU R-12827 | – | – | – | – | KJ363459 | KJ363531 | - | - |
| *P. hispidus* | ZMMU R-12828 | – | – | – | – | KJ363460 | KJ363532 | KJ363420 | KJ363496 |
| *P. hispidus* | ZMMU R-12837 | KF691732 | KF691644 | KF691663 | KF691694 | - | - | - | - |
| *P. incertus* | ZMMU R-13088, ZMMU R-12769, ZMMU R-12673, GB | KF691728 | KF691637 | GQ242206 | KF691690 | KJ363454 | KJ363526 | KJ363415 | KJ363492 |
| *P. interscapularis* | ZMMU R-13358, ZMMU R-12268, GB | KF691704 | KF691620 | AF128517 | KF691671 | KJ363475 | KJ363548 | KJ363436 | KJ363510 |
| *P. kulagini* | ZMMU R-12939-1,2 | KF691735 | KF691641 | KF691664 | KF691696 | KJ363461 | KJ363533 | KJ363421 | KJ363497 |
| *P. kuschakewitschi* | ZMMU R-12516, ZMMU R-12173, GB | KF691727 | KF691643 | GQ242210 | KF691689 | KJ363455 | KJ363527 | KJ363416 | KJ363493 |
| *P. longicaudatus* | ZMMU R-13899-1 | KF691737 | KF691645 | KF691666 | KF691698 | KJ363469 | KJ363542 | KJ363431 | - |
| *P. longicaudatus* | ZMMU R-13899-2 | - | - | - | - | KJ363470 | KJ363543 | KJ363432 | KJ363506 |
| *P. maculatus* | ZMMU R-13029 | KF691707 | KF691623 | KF691650 | KF691674 | KJ363471 | KJ363544 | KJ363433 | - |
| *P. melanurus* 1 | ZMMU R-12332, ZMMU R-12767, GB | KF691725 | KF691636 | GQ242197 | KF691687 | KJ363450 | KJ363523 | KJ363411 | KJ363488 |
| *P. melanurus* 2 | Pfr, ZMMU R-12327, GB | KF691726 | AY053919 | AY396572 | KF691688 | KJ363451 | - | KJ363412 | KJ363489 |
| *P. moltschanovi* | ZMMU R-12942 | KF691730 | KF691638 | KF691662 | KF691691 | KJ363452 | KJ363524 | KJ363413 | KJ363490 |
| *P. mystaceus* 1 | ZMMU R-12261, Pmy2, GB | KF691713 | KF691626 | AF128518 | AY054055 | KJ363478 | KJ363551 | DQ340735 | GQ242237 |
| *P. mystaceus* 2 | ZMMU R-13009 | KF691714 | KF691627 | KF691656 | KF691680 | KJ363477 | KJ363550 | KJ363439 | KJ363513 |
| *P. ocellatus* (= *P. reticulatus*) | ZMMU R-13359, ZMMU R-12272 | KF691709 | KF691624 | KF691652 | KF691676 | KJ363466 | KJ363539 | KJ363428 | KJ363503 |
| *P. ornatus vindumi* | ZMMU R-13026, ZMMU R-13027 | KF691706 | KF691622 | KF691649 | KF691673 | KJ363474 | KJ363547 | KJ363435 | KJ363509 |
| *P. persicus* | ZMMU R-12466, ZMMU R-13243-1,2,3, ZMMU R-12322 | KF691715 | KF691631 | KF691657 | KF691681 | KJ363463 | KJ363536 | KJ363424 | KJ363500 |
| *P. przewalskii* | KIZ 014306 | - | - | - | - | KJ363458 | KJ363530 | KJ363419 | KJ363495 |
| *P. przewalskii* | ZMMU R-8900, GB | KF691734 | AY053941 | AY396586 | AY054061 | - | - | - | - |
| *P. putjatai* | KIZ 020238, ZMMU R-12308, GB | KF691722 | KF691634 | HM629331 | KF691686 | KJ363448 | KJ363521 | KJ363408 | KJ363487 |
| *P. raddei* | ZMMU R-14984, IZIP998 | KF691710 | KF691625 | KF691653 | KF691678 | KJ363467 | KJ363540 | KJ363429 | KJ363504 |
| *P. rossikowi* | ZMMU R-12795 | MG739300 | MG739302 | - | MG739301 | - | - | - | - |
| *P. saidalievi* | ZMMU R-12802-1,2 | KF691717 | KF691629 | KF691659 | KF691682 | KJ363464 | KJ363537 | KJ363425 | KJ363501 |
| *P. scutellatus* | ZMMU R-13020 | KF691712 | KF691619 | KF691655 | KF691677 | KJ363473 | KJ363546 | - | KJ363508 |
| *P. sogdianus* | ZMMU R-13396-1 | - | - | - | - | - | - | KJ363437 | KJ363511 |
| *P. sogdianus* | ZMMU R-13396-2 | - | KF691621 | KF691648 | KF691672 | KJ363476 | KJ363549 | KJ363438 | KJ363512 |
| *P. sogdianus* | ZMMU R-12800 | KF691705 | - | - | - | - | - | - | - |
| *P.* sp. | GB | - | - | - | - | - | - | - | KC551398 |
| *P. strauchi* | ZMMU R-13247, ZMMU R-11386 | KF691711 | – | KF691654 | KF691679 | KJ363468 | KJ363541 | KJ363430 | KJ363505 |
| *P. theobaldi* | ZMMU R-12138 -1,2, , GB | - | - | - | - | KJ363444 | - | KJ363404 | KC551418 |
| *P. theobaldi theobaldi* | ZMMU R-10816, GB | KF691721 | AY053965 | EU275226 | AY054089 | - | - | - | - |
| *P. theobaldi zetangensis* | ZMMU R-12138-2, GB | KF691723 | AY053992 | EU275228 | AY054114 | - | - | - | - |
| *P. varius* | ZMMU R-12524, ZMMU R-13089б GB | HQ543966 | KF691628 | GQ242183 | AY054050 | - | - | KJ363427 | KC551395 |
| *P. versicolor* | ZMMU R-12834, ZMMU R-12178-2, GB | KF691736 | AY053968 | KF691665 | KF691697 | - | KJ363535 | KJ363423 | KJ363499 |
| *P. vlangalii* | KIZ 020062 | KF691719 | KF691642 | KF691660 | KF691685 | KJ363447 | KJ363520 | KJ363407 | KJ363486 |
| *Phrynosoma* | GB | U66224 | U66224 | U66224 | U66224 | JF805850 | JF805850 | JF805850 | FJ356738 |
| *Physignathus cocincinus* | GB | - | - | - | - | JF805830 | JF805830 | JF805830 | JF805830 |
| *Physignathus lesueurii* | GB | - | - | - | - | JF805831 | JF805831 | - | JF805831 |
| *Plica plica* | GB | AB218961 | AB218961 | AB218961 | AB218961 | - | - | - | - |
| *Pogona vitticeps* | GB | NC006922 | NC006922 | NC006922 | NC006922 | JF805832 | JF805832 | JF805832 | JF805832 |
| *Polychrus marmoratus* | GB | NC012839 | NC012839 | NC012839 | NC012839 | JF805852 | JF805852 | JF805852 | JF805852 |
| *Pseudotrapelus sinaitus* | GB | NC013603 | NC013603 | NC013603 | NC013603 | - | - | - | - |
| *Rieppeleon kerstenii* | GB | AB474918 | AB474918 | AB474918 | AB474918 | - | - | - | - |
| *Sauromalus ater* | GB | - | - | - | - | JF805854 | JF805854 | JF805854 | JF805854 |
| *Sceloporus* | GB | NC005960 | NC005960 | NC005960 | NC005960 | JF805855 | JF805855 | JF805855 | GQ494820 |
| *Stellagama stellio* | Lst, ZMMU R-11324 | KF691700 | KF691616 | AF128516 | KF691669 | KJ363443 | KJ363517 | KJ363403 | KJ363483 |
| *Stenocercus* | GB | - | - | - | - | JF805856 | JF805856 | JF805856 | AY662597 |
| *Takydromus sexlineatus* | GB | - | - | - | - | JF805809 | JF805809 | JF805809 | JF805809 |
| *Takydromus tachydromoides* | GB | NC008773 | NC008773 | NC008773 | NC008773 | - | - | - | - |
| *Trapelus* | GB | - | - | - | - | JF805833 | JF805833 | JF805833 | FJ356736 |
| *Trapelus agilis* | Tag | - | - | - | - | - | - | KJ363401 | KJ363480 |
| *Trapelus sanguinolentus* | ZMMU R-12709, ZMMU R-12732, GB | – | AY053997 | GQ242223 | KF691668 | - | - | - | - |
| *Trioceros melleri* | GB | AB474916 | AB474916 | AB474916 | AB474916 | - | - | - | - |
| *Tropidurus plica* | GB | - | - | - | - | JF805857 | JF805857 | JF805857 | - |
| *Uma scoparia* | GB | - | - | - | - | JF805858 | JF805858 | JF805858 | GQ896029 |
| *Uranoscodon superciliosus* | GB | - | - | - | - | JF805859 | JF805859 | JF805859 | - |
| *Uromasticinae* | GB | NC014182 | NC014182 | NC014182 | NC014182 | JF805834 | JF805834 | JF805834 | AY662588 |
| *Uta* | GB | - | - | - | - | JF805861 | JF805861 | JF805861 | GQ896034 |
| *Xenagama batillifera* | GB | AB113825 | AB113825 | AB113825 | AB113825 | - | - | - | - |
| *Xenagama taylori* | GB | DQ008215 | DQ008215 | DQ008215 | DQ008215 | - | - | - | - |
