# Supplementary material for: Cenozoic aridization in Central Eurasia shaped diversification of toad-headed agamas (Phrynocephalus; Agamidae, Reptilia)
Source: PeerJ. 2018 Mar 19;6:e4543. doi: 10.7717/peerj.4543 (PMC5863718; doi:10.7717/peerj.4543)
Supplement: Supplemental Information 18 — Total length (in b.p.), number of conservative (Cons.), variable (Var.) and parsimony-informative (Pars.-Inf.) sites are given (data presented only for the ingroup). [file peerj-06-4543-s018.docx]

| **Gene name - mtDNA** | **Length (b.p.)** | **Cons.** | **Var.** | **Pars.-Inf.** |
| --- | --- | --- | --- | --- |
| ***COI*** | 654 | 386 | 265 | 218 |
| ***Cytb*** | 291 | 153 | 135 | 110 |
| ***ND2*** | 1053 | 426 | 607 | 475 |
| ***ND4*** | 705 | 316 | 377 | 294 |
| **Total**  **Gene name - nuDNA** | **2703** | **1281** | **1384** | **1851** |
| ***AKAP9*** | 1182 | 1042 | 124 | 56 |
| ***NKTR*** | 876 | 612 | 214 | 130 |
| ***BDNF*** | 675 | 610 | 65 | 39 |
| ***RAG-1*** | 1455 | 1283 | 167 | 74 |
| **Total** | **4188** | **3547** | **570** | **1777** |
