# Supplementary material for: Cenozoic aridization in Central Eurasia shaped diversification of toad-headed agamas (Phrynocephalus; Agamidae, Reptilia)
Source: PeerJ. 2018 Mar 19;6:e4543. doi: 10.7717/peerj.4543 (PMC5863718; doi:10.7717/peerj.4543)
Supplement: Supplemental Information 19 — “F,” “L”–forward primer, “R,” “H”–reverse primer. [file peerj-06-4543-s019.docx]

| **Gene** | **Primer name** | **Sequence of 3`–5`** | **Source** |
| --- | --- | --- | --- |
| *COI* | VF1d | TTCTCAACCAACCACAARGAYATYGG | Ivanova *et al.* 2006 |
|  | VR1d | TAGACTTCTGGGTGGCCRAAYCA |  |
|  | R107a | GGCRTGRAGTGTAATAAAGGTGTTGTATA | This study; primer pairs designed for analysis of degraded old DNA from historical collections |
|  | F82 | CCAGGCCAAYACARCGGAGATAC |  |
|  | R268 | TTGATGATATTARGAGTAARAAGAATGATGG |  |
|  | F250 | AAGTTTCTGATTATTACCCCCATC |  |
|  | R372a | GMGAAAATRGTTATRTCTATTGATGG |  |
|  | F354 | CATAGCACACTCAAGCCCATCAATAGA |  |
|  | R490a | AATACGGATCAGACRAATAGYGGTCA |  |
|  | F453 | CATYAACATAACCCCACAYTCAACAA |  |
|  | R580a | CAAAGAATGTTGTRTTTAGGTTTCGGTCTGT |  |
|  | F539 | ATYCCAGTAYTAGCAGCAGCCATTAC |  |
| *ND2* | L4447 | AAGCAGTTGGGCCCATGCCCCAAAAACGG | Wang & Fu 2004 |
|  | H5622 | TATTTTAATTAAAATATCTGAGTTGCA |  |
|  | tRNA-Met | AAGCAGTTGGGCCCATRCC | Macey *et al.* 2000 |
|  | *COI* | AGRGTTCCRATRTCTTTRTGRTT |  |
| *ND4* | ND4 | CACCTATGACTACCAAAAGCTCATGTAGAAGC | Arevalo *et al.* 1994 |
|  | LEU | CATTACTTTTACTTGCACCA |  |
|  | 110R | TTATAATAATTCCTCATGTGGCTATTGC | This study; primer pairs designed for analysis of degraded old DNA from historical collections |
|  | 76F | CCAATAAACATCCCAACCACCTTAA |  |
|  | 180R | CCTACTGAGGAGTAGGCAATAAG |  |
|  | 117F | AATTGCAATAGCCACATGAGGAATT |  |
|  | 240R | GAGGTGCTCCATGGGGTTTGAA |  |
|  | 195F | CTCAGTAGGCCACATAGGACTAG |  |
|  | 331R | ATTCGGGTGTTTGTTCGTTC |  |
|  | 253F | ACCTCCGGGGCAATTATCCT |  |
|  | 428R | TTAGRTTRATTGTTGGGGGTAGTGCTA |  |
|  | 346F | GACCAAACACCCGAATACTAAT |  |
|  | 511R | GGTTGCTCCTGTGGCRGTTA |  |
|  | 452F | AACAATCAACCTAATTGGAGAAATC |  |
|  | 590R | GGGCAGGRCAAATTCCTTTTGARTCTT |  |
|  | 527F | AACCATTACAGCAATCTACTC |  |
| *Cytb* | L14841 | AAAAAGCTTCCATCCAACATCTCAGCATGATGAAA | Pang *et al.* 2003 |
|  | H15149 | AAACTGCAGCCCCTCAGAATGATATTTGTCCTCA |  |
|  | 180R | ATCCGTARTAGATTCCCCGTCC | This study; primer pairs designed for analysis of degraded old DNA from historical collections (the reverse primer, works with L14841 as a forward primer) |
| *RAG-1* | RAG-1-F | CAAAGTGAGACSACTTGGAAAGCC | Shoo *et al.* 2008 |
|  | RAG-1-R | CATTTTTCAAGGGTGGTTTCCACTC |  |
|  | Phryn-670r | GGTTTCTACAGGATCAGCCAGGATA | This study |
|  | Phryn-522f | GTCATGTGAGGAATGTAAAACAAGTGAAC |  |
| *BDNF* | BDNF-F | gACCATCCTTTTCCTKACTATggTTATTTCA | Townsend *et al.* 2008 |
|  | BDNF-R | CTATCTTCCCCTTTTAATggTCAgTgTACAAAC |  |
| *AKAP9* | R1276 | TGCCTGTTCTTGTCCTCCTCTAAATC | Townsend *et al.* 2011 |
|  | F88 | GGTAATACCAGTGAAGACCAAATACAC |  |
|  | f627a | GGAGAACATGGTTATGCAAGCTAAACAG | This study |
|  | r810b | GGAGGKTGTGGATTTGAAGCATC |  |
| *NKTR* | F414 | GGACGGCAAGAAGCAAACGGCAG | Townsend *et al.* 2011 |
|  | R1278a | CGATCGGGTGCGGCTCCTTGAC |  |
|  | r931 | CTACTGCAGCAGCTATGGTGGGAGGTTG | This study |
|  | f686 | GCGAAGGAGAAGTTAACCAGATGGATGCA |  |
