# Supplementary material for: Cenozoic aridization in Central Eurasia shaped diversification of toad-headed agamas (Phrynocephalus; Agamidae, Reptilia)
Source: PeerJ. 2018 Mar 19;6:e4543. doi: 10.7717/peerj.4543 (PMC5863718; doi:10.7717/peerj.4543)
Supplement: Supplemental Information 20 — Node–tree node used for calibration, for node names see Fig. S10; Divergence time given in millions years (Ma); Fossil record–reference on the fossil record used for node calibration; Dataset for analysis–calibration used for mtDNA (mt), nuDNA (nu) or both (mt,nu) datasets. [file peerj-06-4543-s020.docx]

| **Node** | **Divergence time (Ma)** | **Fossil record** | **Dataset for analysis** |
| --- | --- | --- | --- |
| Agamurom | 33.9 | The fossil agamid *Uromastyx europaeus* from the beginning of the Oligocene (33.9 ± 0.1) (Townsend *et al.* 2011). | mt, nu |
| Scelo, Phrynosomatidae | 36.0 | The fossil *Tuberculacerata* from the Medicine Pole Hills in the Chadronian Formation (33.9—38 Mya) of North Dakota (Townsend *et al.* 2011). | mt, nu |
| Acrodonta | 48.6–70.6 | The stem chamaeleonid *Anquingosaurus* from the end of the Lower Eocene (48.6 ± 0.2 Mya), fossils of the stem acrodont iguanian clade Priscagaminae (70.6 ± 0.6 Mya) (Townsend *et al.* 2011). | mt, nu |
| Cham | min 18.0 | Fossil *Chameleo*, but with morphological similarities to *Rhampholeon*, from Rusinga Island, Lake Victoria, Kenya (Rieppel *et al.* 1992) | mt |
| Agamamphi | min 21.0 | The stem fossils from the lineage leading to *Istiurus lesueuriii* dated at 21 Mya (Townsend *et al.* 2011). | mt, nu |
| Brachyloph | 21.0 | The common ancestor of *Dipsosaurus* and its sister taxon from the Lower Miocene (16-23 Mya) (Townsend *et al.* 2011). | nu |
| Iguania | min 55.0 | The stem corytophanid *Suzanniwana patriciana* from the earliest Eocene (approximately 55 Mya) (Townsend *et al.* 2011). | nu |
| Scelouta | min 20.3 | The fossil *Sceloporus* from the Hemingfordian Formation (16.0—20.4 Mya) of Nebraska (Townsend *et al.* 2011). | nu |
