# Supplementary material for: Cenozoic aridization in Central Eurasia shaped diversification of toad-headed agamas (Phrynocephalus; Agamidae, Reptilia)
Source: PeerJ. 2018 Mar 19;6:e4543. doi: 10.7717/peerj.4543 (PMC5863718; doi:10.7717/peerj.4543)
Supplement: Supplemental Information 21 — Geographic regions: AR–Near East and Arabia, MI–Asia Minor and Transcaucasia, KZ–Kazakhstan, northern Caspian and Ciscaucasian deserts, CA–Central Asia, TU–Turan, TI–Tibet, ME–Middle East. Maximum SVL (maxSVL) values are given in mm. Substrate states (Substrate) are classified as: (1) loose sand dunes; (2) sands with non-differentiated proluvial sediments, e.g. gravel or clay; (3) gravel and stone deserts; (4) clay soils and salines; (5) clay soils mixed with gravel; (6) large rocks and cliffs. [file peerj-06-4543-s021.docx]

| **Species** | **Distribution** | | | | | | | **maxSVL (mm)** | **Substrate** |
| --- | --- | --- | --- | --- | --- | --- | --- | --- | --- |
|  | **AR** | **MI** | **TU** | **KZ** | **ME** | **TI** | **CA** |  |  |
| *P. longicaudatus* | 1 | 0 | 0 | 0 | 0 | 0 | 0 | 67 | (2 4) |
| *P. maculatus* | 0 | 0 | 0 | 0 | 1 | 0 | 0 | 91 | (5) |
| *P. melanurus* 1 | 0 | 0 | 0 | 1 | 0 | 0 | 1 | 65 | (1) |
| *P. melanurus* 2 | 0 | 0 | 0 | 0 | 0 | 0 | 1 | 61 | (3) |
| *P. moltschanovi* | 0 | 0 | 1 | 1 | 0 | 0 | 0 | 50 | (2) |
| *P. mystaceus* 1 | 0 | 0 | 1 | 1 | 0 | 0 | 0 | 123 | (1) |
| *P. mystaceus* 2 | 0 | 0 | 1 | 0 | 0 | 0 | 0 | 86 | (1) |
| *P. ocellatus* | 0 | 0 | 1 | 0 | 0 | 0 | 0 | 55 | (4) |
| *P. ornatus vindumi* | 0 | 0 | 0 | 0 | 1 | 0 | 0 | 37 | (1) |
| *P. persicus horvathi* | 0 | 1 | 0 | 0 | 1 | 0 | 0 | 53 | (3 4) |
| *P. przewalskii* | 0 | 0 | 0 | 0 | 0 | 0 | 1 | 72 | (1 2) |
| *P. putjatai* | 0 | 0 | 0 | 0 | 0 | 1 | 0 | 84 | (3) |
| *P. raddei boettgeri* | 0 | 0 | 1 | 0 | 0 | 0 | 0 | 58 | (4) |
| *P. saidalievi* | 0 | 0 | 1 | 0 | 0 | 0 | 0 | 73 | (3 4) |
| *P. scutellatus* | 0 | 0 | 0 | 0 | 1 | 0 | 0 | 56 | (4 5) |
| *P. sogdianus* | 0 | 0 | 1 | 0 | 0 | 0 | 0 | 47 | (1) |
| *Phrynocephalus* sp. | 0 | 0 | 0 | 0 | 0 | 0 | 1 | 55 | (2) |
| *P. strauchi* | 0 | 0 | 1 | 0 | 0 | 0 | 0 | 52 | (2 3) |
| *P. theobaldi* | 0 | 0 | 0 | 0 | 0 | 1 | 0 | 57 | (3) |
| *P. varius* | 0 | 0 | 0 | 1 | 0 | 0 | 1 | 51 | (2 5) |
| *P. versicolor* | 0 | 0 | 0 | 0 | 0 | 0 | 1 | 60 | (2 3) |
| *P. vlangalii* | 0 | 0 | 0 | 0 | 0 | 1 | 0 | 77 | (2 3) |
| *P. kushakewitschii* | 0 | 0 | 0 | 1 | 0 | 0 | 0 | 60 | (2 3) |
| *P. kulagini* | 0 | 0 | 0 | 0 | 0 | 0 | 1 | 53 | (2 4) |
| *P. alpherakii* | 0 | 0 | 0 | 1 | 0 | 0 | 0 | 51 | (2 3) |
| *P. arabicus* | 1 | 0 | 0 | 0 | 0 | 0 | 0 | 58 | (1) |
| *P. axillaris* | 0 | 0 | 0 | 0 | 0 | 1 | 1 | 63 | (1 2 4) |
| *P. erythrurus* | 0 | 0 | 0 | 0 | 0 | 1 | 0 | 53 | (3) |
| *P. forsythii* | 0 | 0 | 0 | 0 | 0 | 1 | 0 | 58 | (1 2) |
| *P. frontalis* | 0 | 0 | 0 | 0 | 0 | 0 | 1 | 57 | (1 2) |
| *P. guttatus* | 0 | 0 | 1 | 1 | 0 | 0 | 0 | 50 | (2 5) |
| *P. helioscopus* | 0 | 0 | 1 | 1 | 0 | 0 | 0 | 52 | (4 5) |
| *P. hispidus* 1 | 0 | 0 | 0 | 0 | 0 | 0 | 1 | 55 | (2 3 5) |
| *P. hispidus* 2 | 0 | 0 | 0 | 0 | 0 | 0 | 1 | 56 | (2 3) |
| *P. incertus* | 0 | 0 | 0 | 1 | 0 | 0 | 0 | 60 | (2 5) |
| *P. interscapularis* | 0 | 0 | 1 | 0 | 0 | 0 | 0 | 40 | (1) |
| *Agama agama* | - | - | - | - | - | - | - | 140 | (6) |
| *Paralaudakia caucasia* | - | - | - | - | - | - | - | 177 | (6) |
| *Paralaudakia himalayana* | - | - | - | - | - | - | - | 115 | (6) |
| *Paralaudakia lehmanni* | - | - | - | - | - | - | - | 150 | (6) |
| *Stellagama stellio* | - | - | - | - | - | - | - | 148 | (6) |
| *Trapelus agilis* | - | - | - | - | - | - | - | 116 | (2 3) |
| *Bufoniceps laungwalaensis* | - | - | - | - | - | - | - | 69 | (1) |
| *Pseudotrapelus sinaitus* | - | - | - | - | - | - | - | 100 | (6) |
| *Xenagama batillifera* | - | - | - | - | - | - | - | 86 | (3) |
| *Laudakia nupta* | - | - | - | - | - | - | - | 172 | (6) |
