# Supplementary material for: Cenozoic aridization in Central Eurasia shaped diversification of toad-headed agamas (Phrynocephalus; Agamidae, Reptilia)
Source: PeerJ. 2018 Mar 19;6:e4543. doi: 10.7717/peerj.4543 (PMC5863718; doi:10.7717/peerj.4543)
Supplement: Supplemental Information 22 — Numbers encode: (1) loose sand dunes; (2) sands with non-differentiated proluvial sediments, e.g. gravel or clay; (3) gravel and stone deserts; (4) clay soils and salines; (5) clay soils mixed with gravel; (6) large rocks and cliffs; see Fig. 7. [file peerj-06-4543-s022.docx]

**Substrate: 1 2 3 4 5 6**

**1** - 1 2 2 2 2

**2** 1 - 1 1 2 2

**3** 2 1 - 1 2 2

**4** 2 1 1 - 1 2

**5** 2 2 2 1 - 2

**6** 2 2 2 2 2 -
