# Supplementary material for: Cenozoic aridization in Central Eurasia shaped diversification of toad-headed agamas (Phrynocephalus; Agamidae, Reptilia)
Source: PeerJ. 2018 Mar 19;6:e4543. doi: 10.7717/peerj.4543 (PMC5863718; doi:10.7717/peerj.4543)
Supplement: Supplemental Information 23 — H0–test hypothesis (analysis as a separate marker), H1–original analysis (analysis within the concatenated alignment); AU–AU-test value. [file peerj-06-4543-s023.docx]

| **Gene\Topological hypothesis** | **H_1_ (final alignment)** | **H_0_ (separate marker)** |
| --- | --- | --- |
| ***AKAP9*** | 0.11 | 0.89 |
| ***NKTR*** | 0.71 | 0.29 |
| ***BDNF*** | 0.0 | 0.99 |
| ***RAG-1*** | 0.76 | 0.0 |
