# Supplementary material for: Cenozoic aridization in Central Eurasia shaped diversification of toad-headed agamas (Phrynocephalus; Agamidae, Reptilia)
Source: PeerJ. 2018 Mar 19;6:e4543. doi: 10.7717/peerj.4543 (PMC5863718; doi:10.7717/peerj.4543)
Supplement: Supplemental Information 24 — H0–test hypothesis, H1–original topology; nu_vs_mt–testing nuclear topology on mitochondrial dataset, mt_vs_nu–testing mitochondrial topology on nuclear dataset; AU–AU-test value. [file peerj-06-4543-s024.docx]

| **DNA dataset** | **Hypothesis** | | **AU** |
| --- | --- | --- | --- |
| nu_vs_mt | C is basal clade | H0 | 0.217 |
| nu_vs_mt | A + B + C + D + E as on Fig. 3 | H1 | 0.783 |
| nu_vs_mt | B + C are basal | H0 | 0.277 |
| nu_vs_mt | A + B + C + D + E as on Fig. 3 | H1 | 0.723 |
| nu_vs_mt | D + (F–J) | H0 | 0.217 |
| nu_vs_mt | D + (A–C) as on Fig. 3 | H1 | 0.815 |
| nu_vs_mt | D + H | H0 | 0.000 |
| nu_vs_mt | D + (A–C) as on Fig. 3 | H1 | 0.670 |
| nu_vs_mt | E + I + J | H0 | 0.000 |
| nu_vs_mt | A + B + C + D + E as on Fig. 3 | H1 | 0.993 |
| nu_vs_mt | B is basal | H0 | 0.518 |
| nu_vs_mt | A + B + C + D + E as on Fig. 3 | H1 | 0.482 |
| nu_vs_mt | strauchi+ocellatus | H0 | 0.752 |
| nu_vs_mt | strauchi+ (H - J) as on Fig. 3 | H1 | 0.248 |
| nu_vs_mt | Complete nu topology (only resolved nodes) | H0 | 0.000 |
| nu_vs_mt | Complete mt topology (only resolved nodes) | H1 | 0.950 |
| nu_vs_mt | A+B+C+D | H0 | 0.000 |
| nu_vs_mt | as on Fig. 2 | H1 | 0.230 |
| nu_vs_mt | (((F + G + H + I +J)+E)+A) | H0 | 0.280 |
| nu_vs_mt | as on Fig. 2 | H1 | 0.720 |
| mt_vs_nu | A + B + C | H0 | 0.990 |
| mt_vs_nu | as on Fig. 2 | H1 | 0.990 |
| mt_vs_nu | D + F + H | H0 | 0.000 |
| mt_vs_nu | as on Fig. 2 | H1 | 0.996 |
| mt_vs_nu | A + B + C + D + E | H0 | 0.000 |
| mt_vs_nu | as on Fig. 2 | H1 | 0.958 |
| mt_vs_nu | ocellatus+ F | H0 | 0.857 |
| mt_vs_nu | as on Fig. 2 | H1 | 0.143 |
| mt_vs_nu | (A-E) + (F-J) | H0 | 0.830 |
| mt_vs_nu | as on Fig. 2 | H1 | 0.169 |
| mt_vs_nu | Complete mt topology (only resolved nodes) | H0 | 0.000 |
| mt_vs_nu | Complete nu topology (only resolved nodes) | H1 | 0.990 |
| mt_vs_nu | Complete mt topology (only resolved nodes) | H0 | 0.000 |
| mt_vs_nu | Complete nu topology (only resolved nodes) | H1 | 0.990 |
| mt_vs_nu | D + F + H | H0 | 0.000 |
| mt_vs_nu | as on Fig. 3 | H1 | 0.230 |
| mt_vs_nu | (((F + G + H + I +J)+E)+A) | H0 | 0.490 |
| mt_vs_nu | as on Fig. 3 | H1 | 0.500 |
