# Supplementary material for: Cenozoic aridization in Central Eurasia shaped diversification of toad-headed agamas (Phrynocephalus; Agamidae, Reptilia)
Source: PeerJ. 2018 Mar 19;6:e4543. doi: 10.7717/peerj.4543 (PMC5863718; doi:10.7717/peerj.4543)
Supplement: Supplemental Information 25 — For node names see Supplementary Figure S1. Estimated age is given in (Ma) for nuDNA and mtDNA datasets; results of ML biogeographic area reconstruction in Lagrange (Ancestral Area) is given for nuDNA topology only. For biogeographic areas definition see Fig. 6 and Supplementary File 2. [file peerj-06-4543-s025.docx]

| **node** | **Estimated age (Ma), nuDNA** | | **Estimated age (Ma), mtDNA** | | **Ancestral Area, nuDNA** | |
| --- | --- | --- | --- | --- | --- | --- |
|  | **mean** | **95% CI** | **mean** | **95% CI** | **split** | **relative probability** |
| **ACRODONTA** | 68.09 | 63.74–70.60 | 67.4 | 58.39–113.02 | — | — |
| **AGAMAMPHI** | 52.75 | 47.11–58.20 | 53.4 | 34.14–63.02 | — | — |
| **AGAMDRACO** | 47.05 | 41.25–52.0 | 49.6 | 30.00–60.05 | — | — |
| **AGAMINAE** | 28.85 | 24.09–33.21 | 38.9 | 23.15–50.1 | — | — |
| **AGAMLEIO** | 61.26 | 55.72–66.25 | — | — | — | — |
| **AGAMTRAP** | 22.69 | 17.84–26.78 | 33.7 | 20.55–46.5 | — | — |
| **AGAMUROM** | 65.88 | 60.27–70.60 | — | — | — | — |
| **AMPHIBOL** | 19.00 | 14.43–23.85 | 46.6 | 24.82–60.39 | — | — |
| **ARLONG** | 2.07 | 1.01–3.30 | 5.4 | 1.69–13.38 | — | — |
| **ARMAC** | 4.87 | 2.70–7.09 | 9.2 | 4.39–17.89 | — | — |
| **BRACHYLO** | 21.43 | 19.63–23.37 | — | — | — | — |
| **CHAMAE** | 27.14 | 18.40–36.32 | 38.4 | 21.39–54.92 | — | — |
| **DRACO** | 28.82 | 22.53–35.60 | 27.0 | 10.28–44.29 | — | — |
| **GUTTATUS** | 3.63 | 1.88–5.29 | 5.0 | 3.08–8.78 | MA\|MA | 0.63 |
| **GUTVERS** | 4.73 | 3.51–5.97 | 6.5 | 3.98–10.92 | MA\|CA | 0.58 |
| **HELIO** | 3.46 | 2.14–4.94 | 5.6 | 2.45–10.58 | TU\|TU | 0.53 |
| **HELPERS** | 6.45 | 4.56–8.34 | 8.6 | 4.87–14.14 | ME\|TU | 0.67 |
| **HELRAD** | 7.28 | 5.49–9.10 | 11.2 | 7.12–17.51 | TU\|TU | 0.64 |
| **HISP** | 2.68 | 1.04–4.51 | 2.6 | 0.87–5.79 | CA\|CA | 0.99 |
| **IGUANIA** | 60.54 | 55.00–67.28 | 52.4 | 38.15–77.7 | — | — |
| **INT** | 2.69 | 1.52–3.96 | 1.7 | 0.53–4.98 | TU\|TU | 0.89 |
| **MICROPHR** | 7.65 | 5.66–9.74 | 12.3 | 5.17–22.81 | TU\|ME | 0.51 |
| **MICRSCUT** | 9.79 | 7.64–12.10 | — | — | ME\|ME | 0.69 |
| **MYST** | 2.17 | 0.90–3.56 | 3.7 | 1.08–8.88 | TU\|TU | 0.49 |
| **MYSTAX** | — | — | 10.4 | 5.66–16.8 | — | — |
| **PARALAU** | 5.51 | 3.06–8.22 | 14.5 | 6.38–26.23 | — | — |
| **PARAPAMIR** | 3.39 | 1.45–5.37 | — | — | — | — |
| **PHEAST** | 8.97 | 7.37–10.87 | — | — | TU\|TU+CA | 0.50 |
| **PHLAU** | — | — | 33.2 | 19.92–45.69 | — | — |
| **PHR1** | — | — | 17.6 | 10.68–24.97 | — | — |
| **PHR2** | — | — | 14.3 | 8.98–20.65 | — | — |
| **PHR3** | — | — | 13.2 | 8.39–19.44 | — | — |
| **PHRYNOAL** | 14.76 | 12.01–17.47 | 19.3 | 12.20–28.90 | TU+ME\|TU | 0.27 |
| **PHRYNOSO** | 36.32 | 34.64–37.96 | — | — | — | — |
| **PHRYNOUMA** | 31.89 | 30.30–33.56 | — | — | — | — |
| **PHWEST** | 11.32 | 9.08–13.64 | — | — | ME\|TU | 0.38 |
| **RADDEI** | 4.63 | 2.70–6.62 | 3.9 | 1.2–8.7 | TU\|TU | 0.98 |
| **SAND** | 10.07 | 8.02–12.31 | — | — | ME\|ME | 0.66 |
| **SCELOUTA** | 20.24 | 18.34–22.10 | — | — | — | — |
| **TIBET** | 3.86 | 2.66–5.38 | 6.4 | 3.19–13.4 | TI\|TI | 0.89 |
| **VERS** | 2.55 | 1.51–3.59 | 4.3 | 2.19–7.51 | CA\|CA | 0.99 |
| **WESTTIBET** | 13.50 | 11.00–16.16 | — | — | ME\|TU+ME | 0.38 |
